# Supplementary material for: Multiplexed nanomaterial-assisted laser desorption/ionization for pan-cancer diagnosis and classification
Source: Nat Commun. 2022 Feb 1;13:617. doi: 10.1038/s41467-021-26642-9 (PMC8807648; doi:10.1038/s41467-021-26642-9)
Supplement: Supplementary file 1 — Supplementary Information [file 41467_2021_26642_MOESM1_ESM.pdf]

Supplementary Materials for

Multiplexed Nanomaterial-Assisted Laser Desorption/Ionization for Pan-cancer Diagnosis and Classification

Hua Zhang[1,15], Lin Zhao[2,15], Jingjing Jiang[2,15], Jie Zheng[3,15], Li Yang[1], Yanyan Li[1], Jian Zhou[4], Tianshu Liu[5], Jianmin Xu[6], Wenhui Lou[6], Weige Yang[6], Lijie Tan[7], Weiren Liu[4], Yiyi Yu[5], Meiling Ji[6], Yaolin Xu[6], Yan Lu[2], Xiaomu Li[2], Zhen Liu[8], Rong Tian[8], Cheng Hu[1], Shumang Zhang[1], Qinsheng Hu[9], Yangdong Deng[10], Hao Ying[11], Sheng Zhong[3], Xingdong Zhang[1], Yunbing Wang[1]*, Hua Wang[12]*, Jingwei Bai[8]*, Xiaoying Li[2]*, Xiangfeng Duan[13,14]

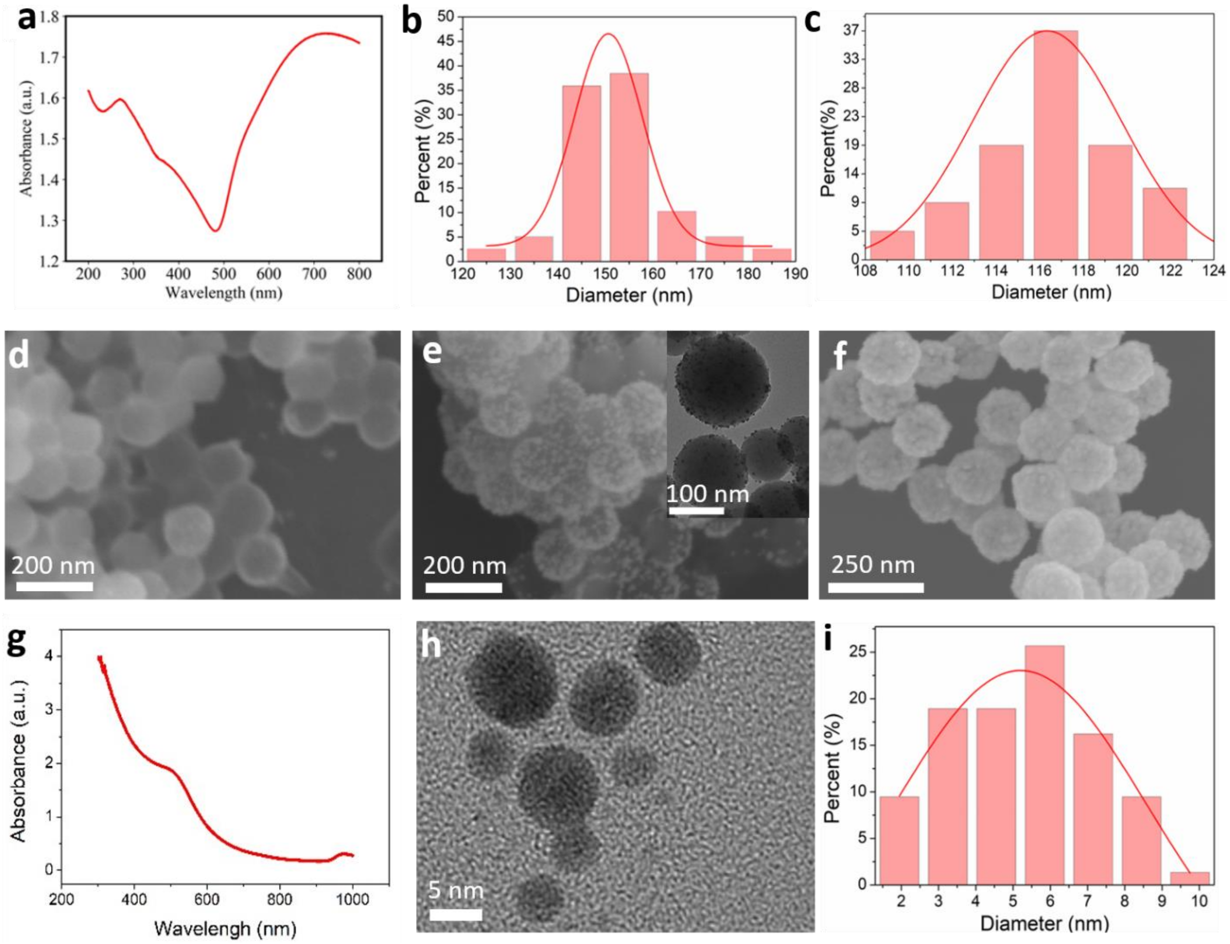

Supplementary Figure 1. The characterization of gold nanoshell nanoparticles. a, UV absorption of gold nanoshell nanoparticles with plasmonic peak around 680nm and strong UV adsorption at UV region which may contributed to the energy conversion from UV laser excitation; b and c are the statistics of size distributions for as synthesized gold nanoshells of about 150 nm and the original silica nanoparticles of about 114 nm before Au shell coating, indicating the shell thickness of 17nm. d, e, f are the SEM pictures of silica nanoparticles, silica nanoparticles with gold nanoseeds attached (with TEM insert) and Au nanoshell nanoparticles (n⩾3 randomly selected); these demonstrate that the growth of the nanoshell initiated from multiple seeds that may explain the rough surface of the as formed Au nanoshells. g, UV absorption of gold nanoseeds; h, TEM characterization of gold nanoseeds (n⩾3 randomly selected), and i, size distribution of gold nanoseeds measured by TEM; these evidences demonstrate the size of the Au nanoseeds which initiated the shell formation are less than 10nm.

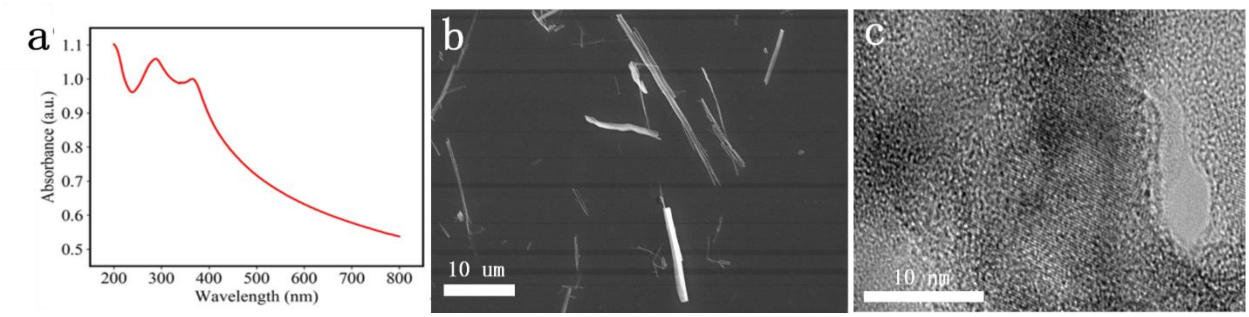

Supplementary Figure 2. The characterization of porous silicon nanowires. a, UV-vis absorption of porous silicon nanowires dispersed in DI water; the absorption in UV region indicates small grains of the nanowires. b, SEM of dispersed silicon wires (n⩾3 randomly selected) by sonicating the etched wafer in water. The SEM sample was prepared by simply drying the silicon wire solution on SEM substrate. As seen from the SEM, the typical diameters of the wires are around ~100-200 nm, the length is on the order of 10 um depending on the etching time. These wires dispersed well in pure water solution without the help of surfactant, indicating the hydrophilic surface nature of the wires. c, the high magnification TEM of porous silicon nanowires (n⩾3 randomly selected) showing 5-10nm pores which explained above UV adsorption, and the edge amorphous structure indicate surface oxide coverage from the H_2O_2 oxidation, which can render the surface hydrophilic. surface hydrophilic properties.

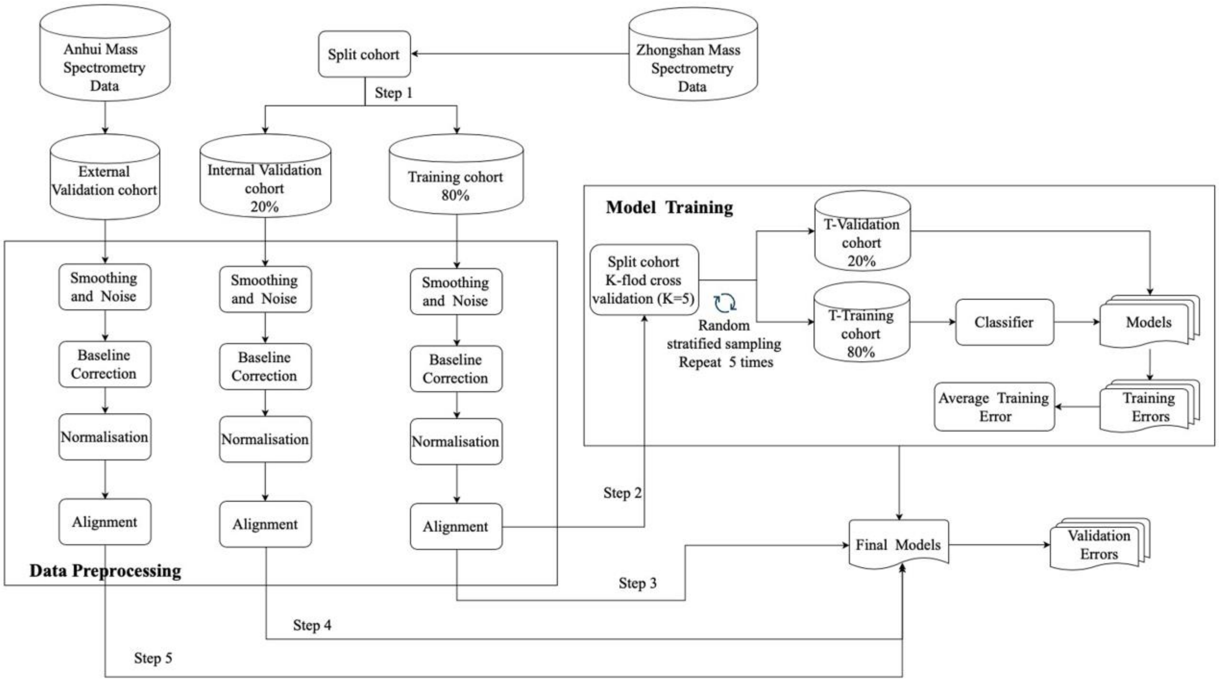

Supplementary Figure 3. Flow diagram for data analysis.

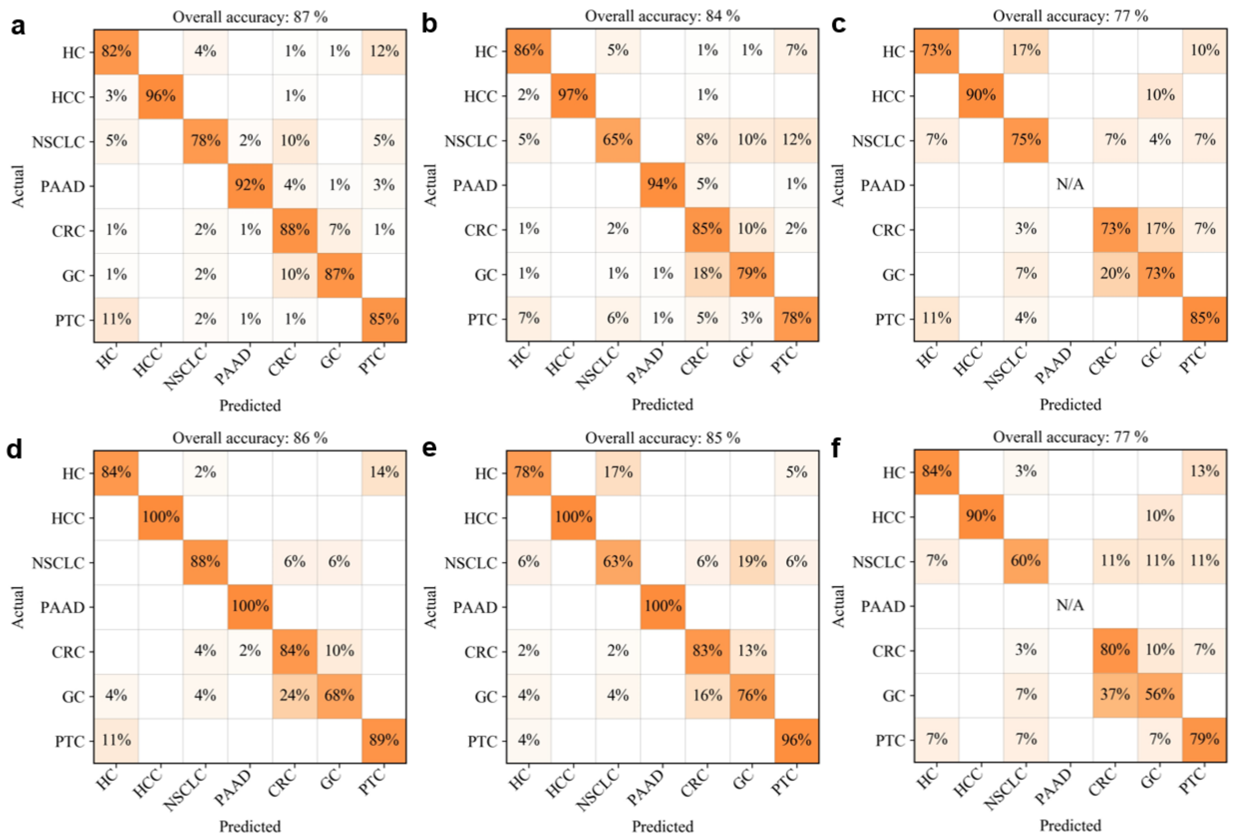

Supplementary Figure 4. Confusion matrix summarizing the cancer classification results in the training cohort (a), the internal (b) and external (c) validation cohort of the GNS-assisted SVM model. Confusion matrix summarizing the cancer classification results in the training cohort (d), the internal (e) and external (f) validation cohort of the SiNW-assisted SVM model.

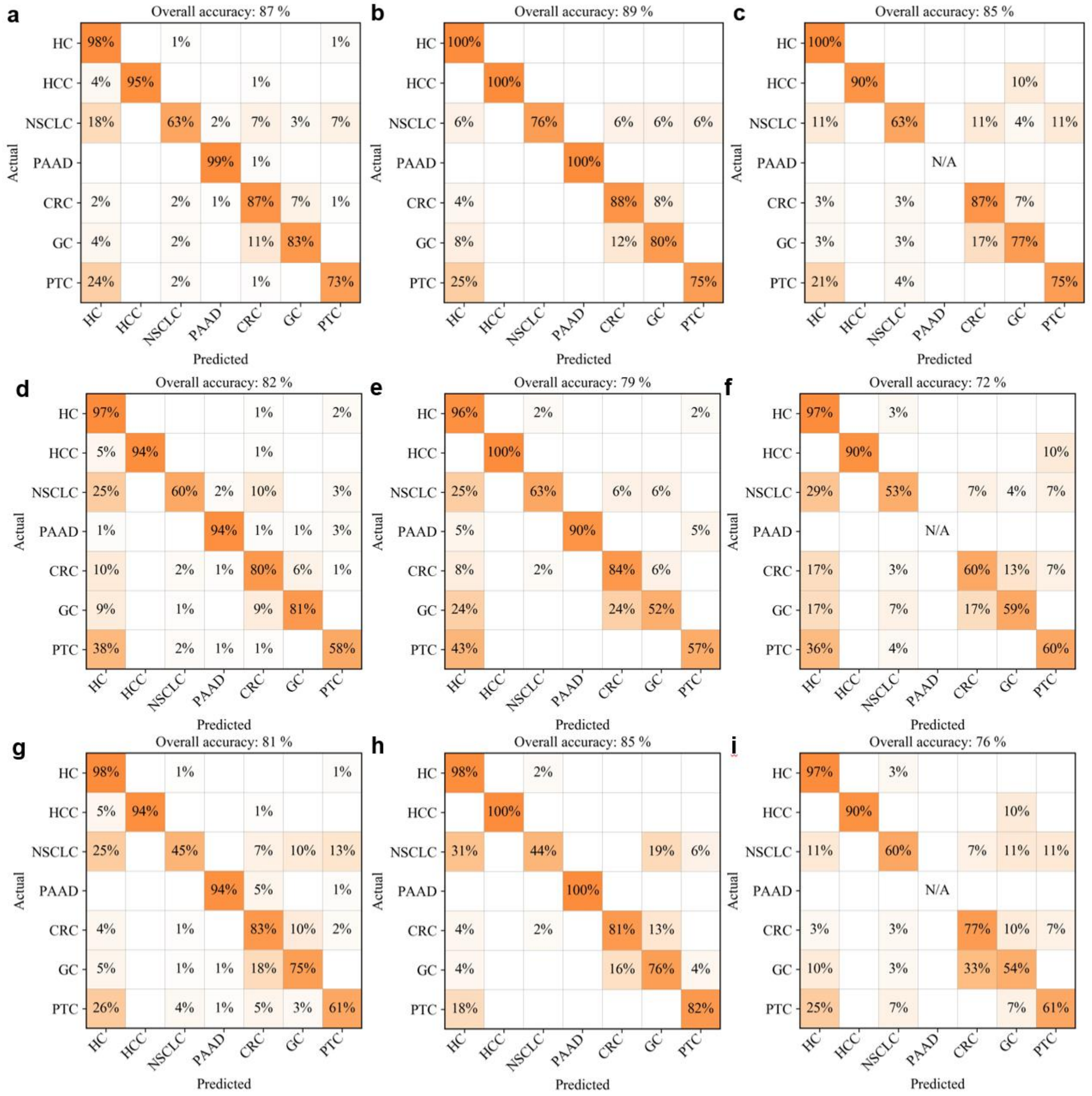

Supplementary Figure 5. Confusion matrix summarizing the cancer classification results with controlled specificity in training cohort (a), internal (b) and external (c) validation cohort of fusion model. Confusion matrix summarizing the cancer classification results with controlled specificity in training cohort (d), internal (e) and external (f) validation cohort of GNS-assisted SVM model. Confusion matrix summarizing the cancer classification results with controlled specificity in the training cohort (g), the internal (h) and external (i) validation cohort of SiNW-assisted SVM model.

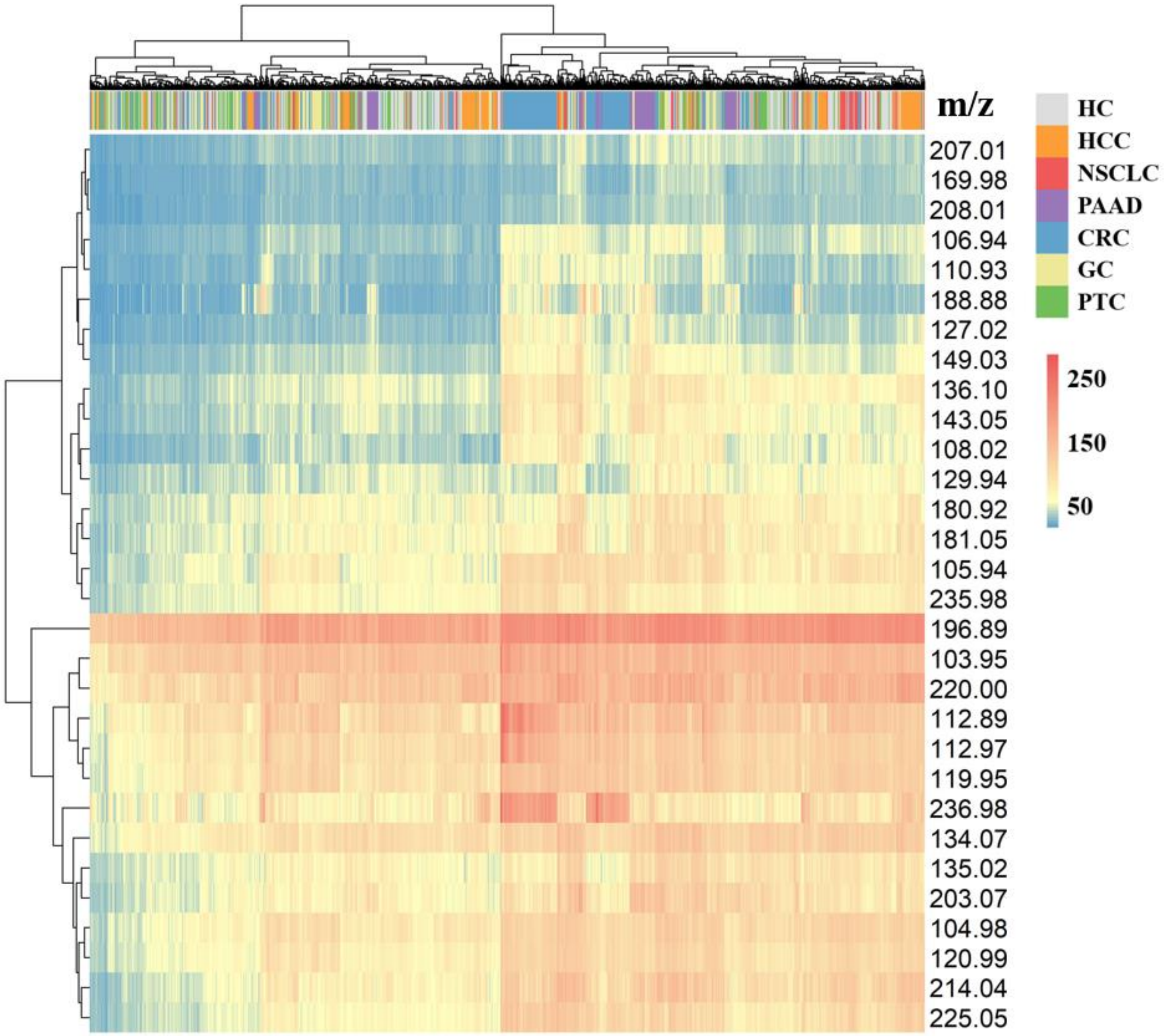

Supplementary Figure 6. Heatmaps based on top 30 features chosen by SVM recursive feature elimination (SVM-RFE), show the difference among 6 types of cancer patients and healthy controls with GNS-assisted LDI. Red represents high, and blue represents low.

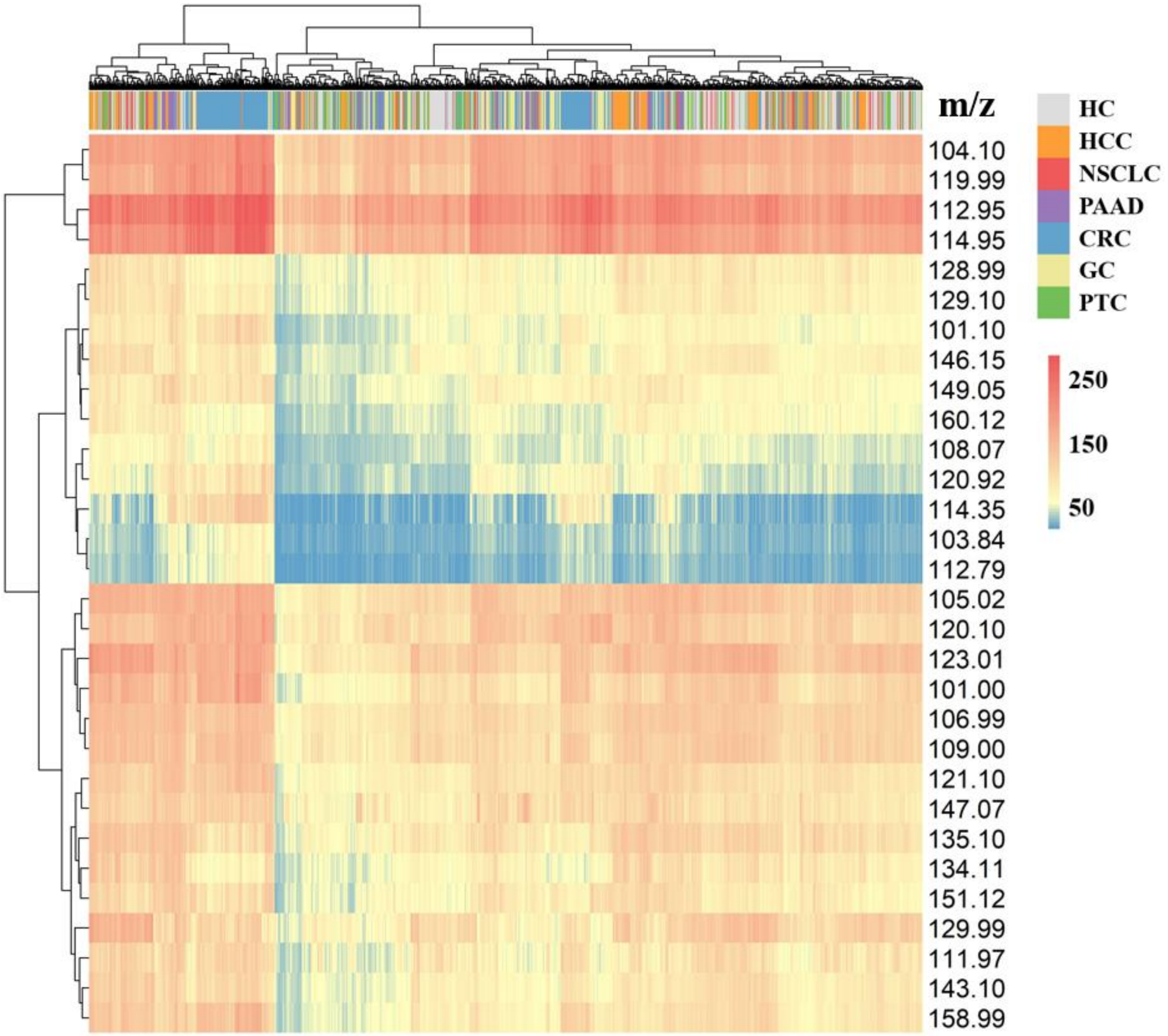

Supplementary Figure 7. Heatmaps based on top 30 features chosen by SVM recursive feature elimination (SVM-RFE), show the difference among 6 types of cancer patients and healthy controls with SiNW-assisted LDI. Red represents high, and blue represents low.

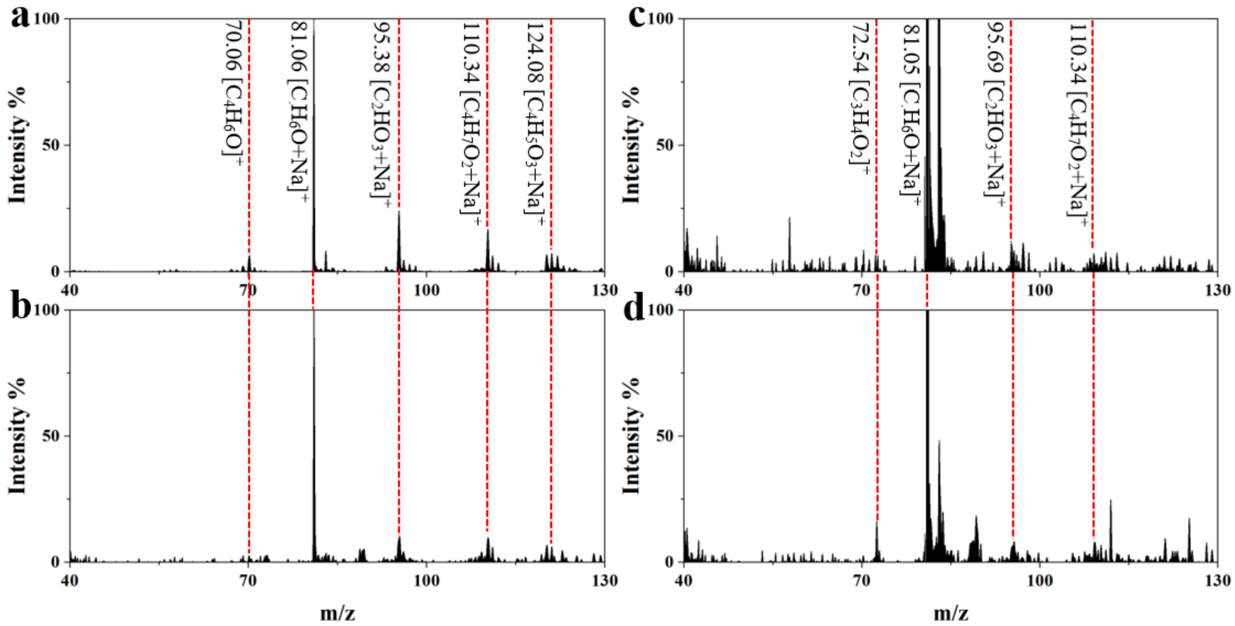

Supplementary Figure 8. MS/MS data of Na^+ adducted 2-oxovaleric acid. MS/MS of 2-oxovaleric acid at m/z of 138.98 for $[M+Na]^+$ in (a) standard sample mixed with GNS, (b) serum sample mixed with GNS, (d) standard sample mixed with SiNW and (d) serum sample mixed with SiNW.

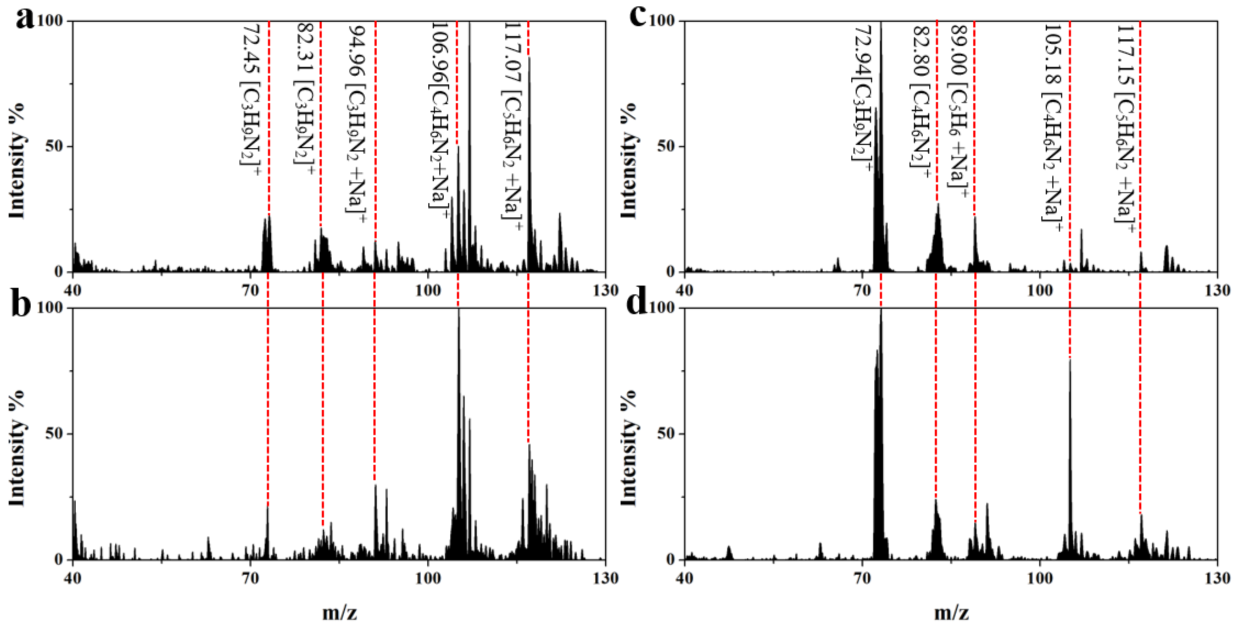

Supplementary Figure 9. MS/MS data of Na^+ adducted histamine. MS/MS of histamine at m/z of 134.01 for $[M+Na]^+$ in (a) standard sample mixed with GNS, (b) serum sample mixed with GNS, (d) standard sample mixed with SiNW and (d) serum sample mixed with SiNW.

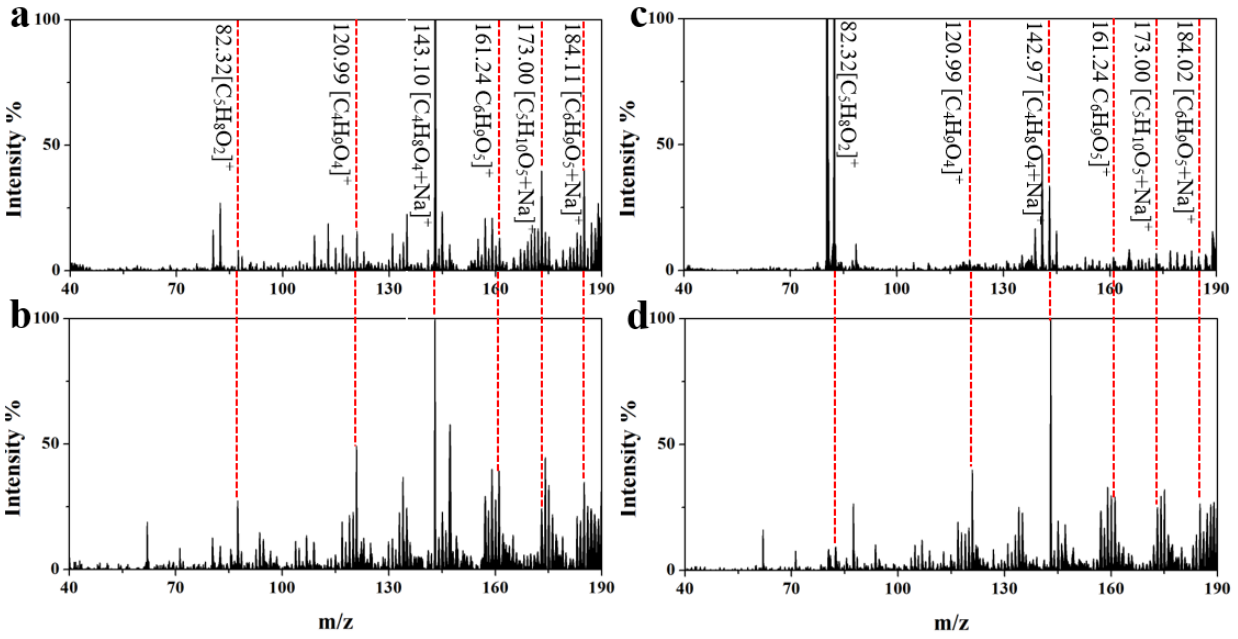

Supplementary Figure 10. MS/MS data of Na^+ adducted glucose. MS/MS of glucose at m/z of 203.09 for $[M+Na]^+$, and 219.09 for $[M+K]^+$ in (a) standard sample mixed with GNS, (b) serum sample mixed with GNS, (d) standard sample mixed with SiNW and (d) serum sample mixed with SiNW.

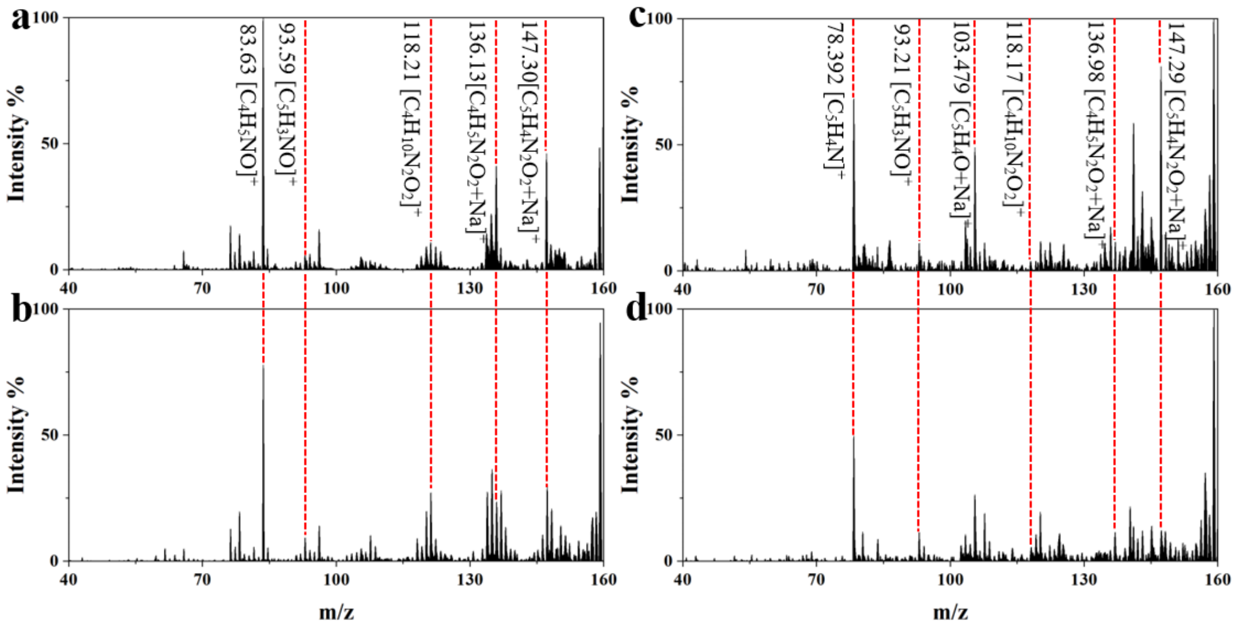

Supplementary Figure 11. MS/MS data of Na^+ adducted 5-hydroxymethyluracil. MS/MS of 5-hydroxymethyluracil at m/z of 164.98 for $[M+Na]^+$ in (a) standard sample mixed with GNS, (b) serum sample mixed with GNS, (d) standard sample mixed with SiNW and (d) serum sample mixed with SiNW.

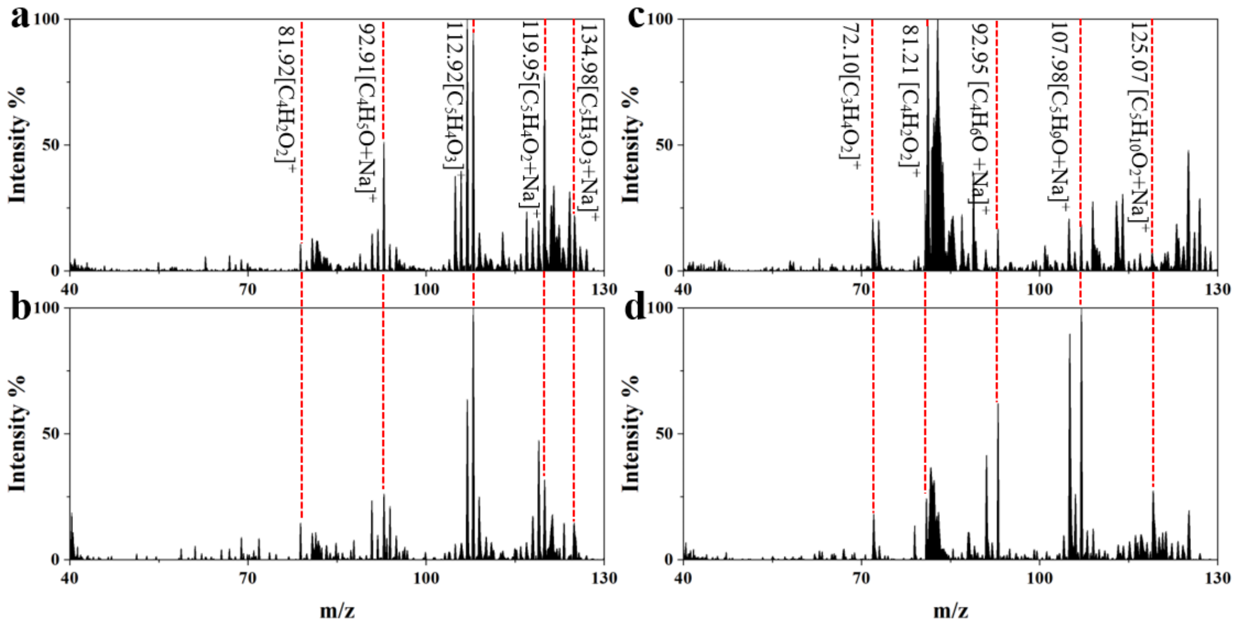

Supplementary Figure 12. MS/MS data of Na^+ adducted 2-Furoic acid. MS/MS of 2-Furoic acid at m/z of 135.02 for $[M+Na]^+$ in (a) standard sample mixed with GNS, (b) serum sample mixed with GNS, (d) standard sample mixed with SiNW and (d) serum sample mixed with SiNW.

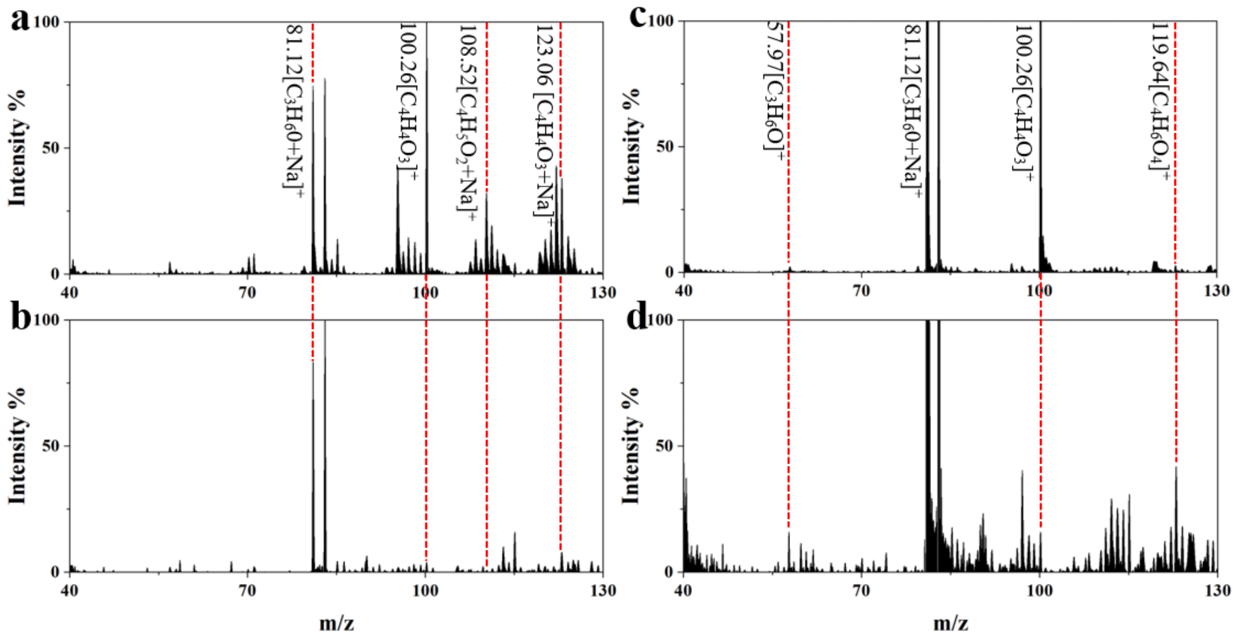

Supplementary Figure 13. MS/MS data of Na^{+} adducted methylmalonic acid. MS/MS of methylmalonic acid at m/z of 140.94 for $[M+Na]^{+}$ in (a) standard sample mixed with GNS, (b) serum sample mixed with GNS, (d) standard sample mixed with SiNW and (d) serum sample mixed with SiNW.

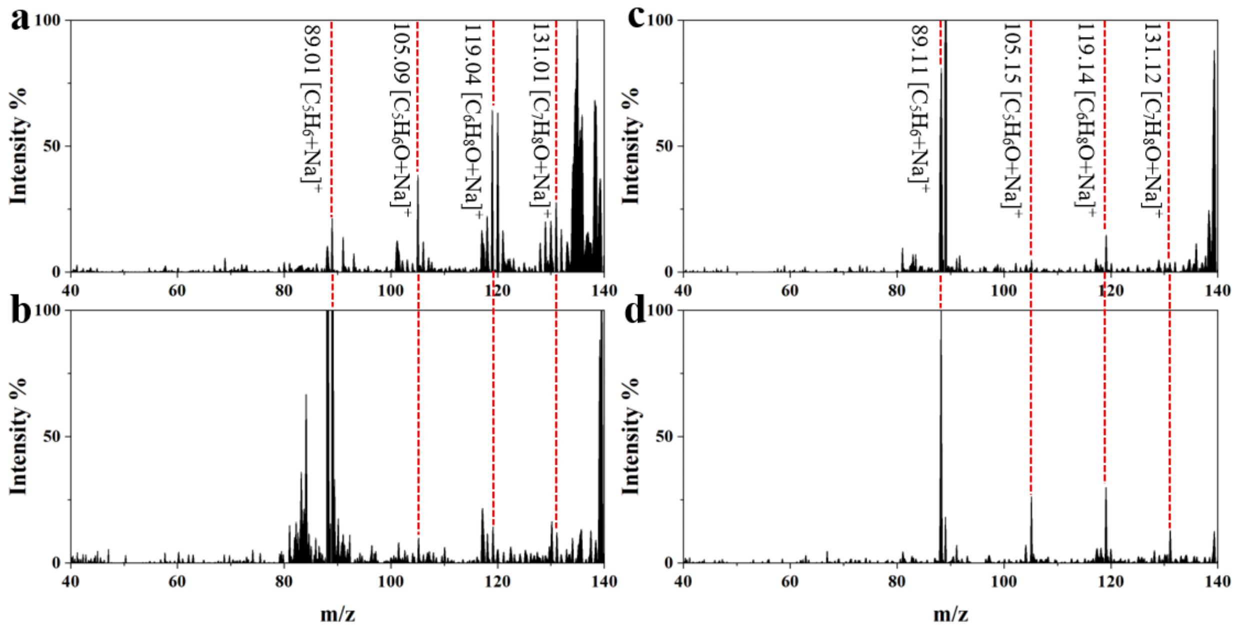

Supplementary Figure 14. MS/MS data of Na^+ adducted methylcatechol. MS/MS of methylcatechol at m/z of 147.01 for $[M+Na]^+$ in (a) standard sample mixed with GNS, (b) serum sample mixed with GNS, (d) standard sample mixed with SiNW and (d) serum sample mixed with SiNW.

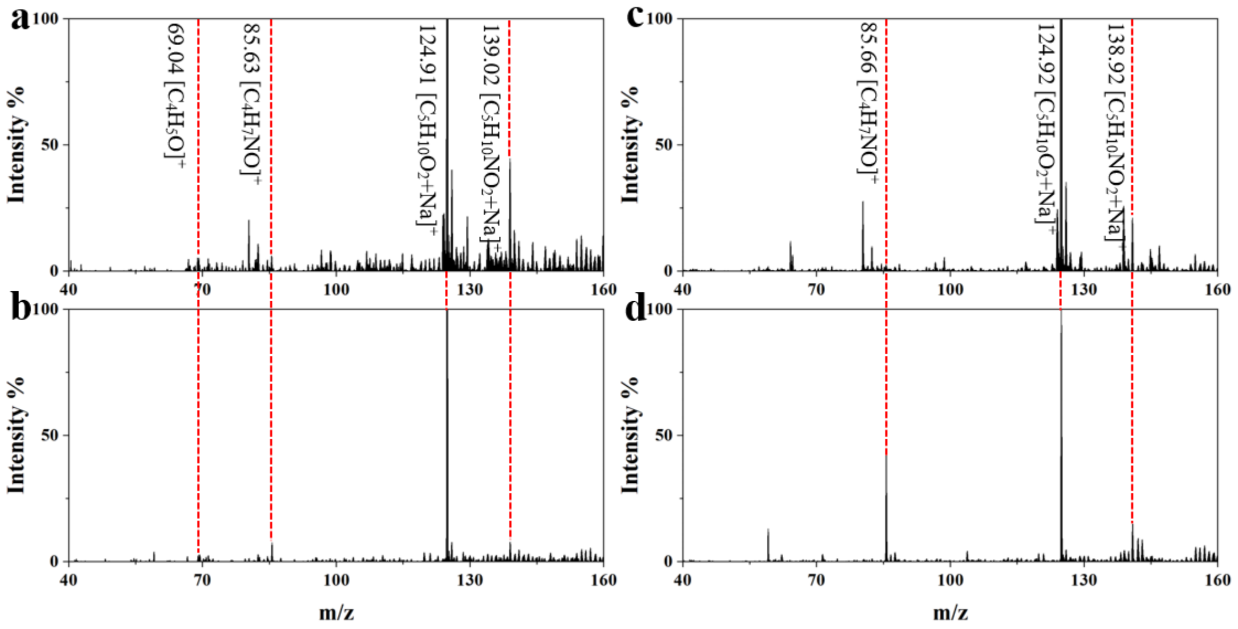

Supplementary Figure 15. MS/MS data of Na^+ adducted L-carnitine. MS/MS of L-carnitine at m/z of 184.01 for $[M+Na]^+$ in (a) standard sample mixed with GNS, (b) serum sample mixed with GNS, (d) standard sample mixed with SiNW and (d) serum sample mixed with SiNW.

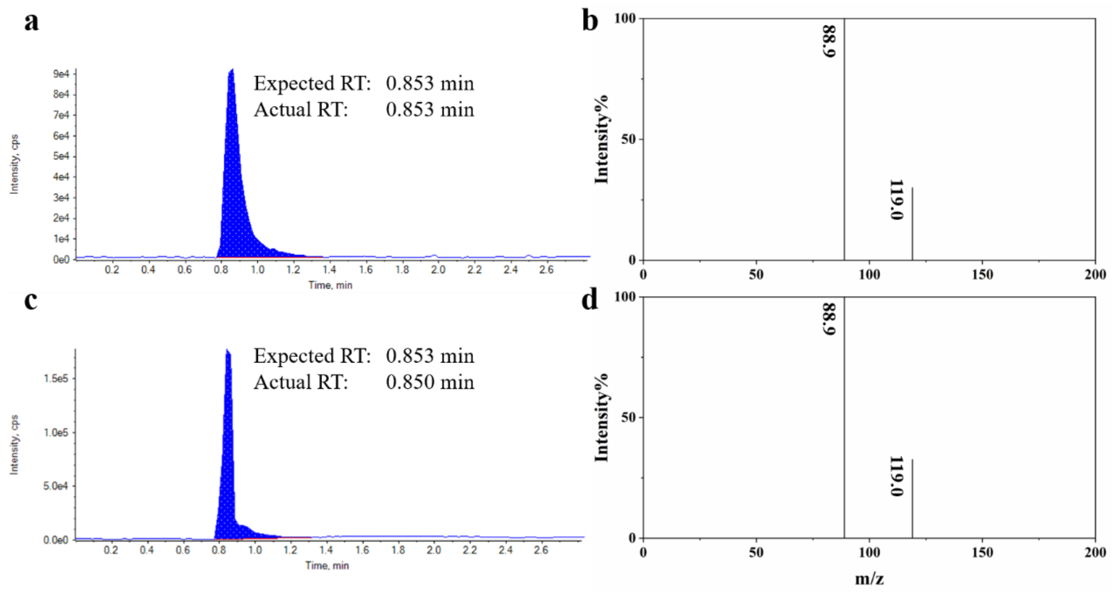

e MRM Acquisition Parameter List

Analytes	Q1 Mass（Da）	Q3 Mass（Da）	DP(V)	CE(V)	CXP(V)
Glucose	179.0	119.0	-58	-10	-7
	179.0	88.9	-48	-12	-5

Supplementary Figure 16. LC MS/MS of glucose. a) retention time in standard sample; b) MS/MS date in standard sample; c) retention time in serum sample; d) MS/MS data in serum sample; e) MRM acquisition parameter list.

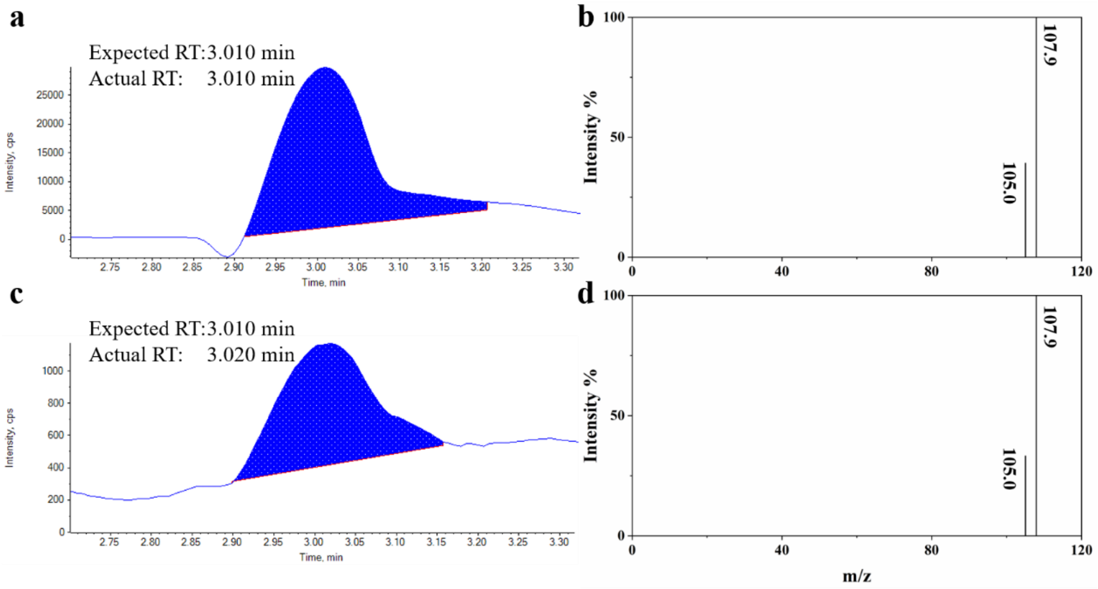

e

MRM Acquisition Parameter List

Analytes	Q1 Mass (Da)	Q3 Mass (Da)	DP(V)	CE(V)	CXP(V)
Methylcatechol	123.0	107.9	-100	-24	-6
	123.0	105.0	-100	-25	-7

Supplementary Figure 17. LC MS/MS of methylcatechol. a) retention time in standard sample; b) MS/MS date in standard sample; c) retention time in serum sample; d) MS/MS data in serum sample; e) MRM acquisition parameter list.

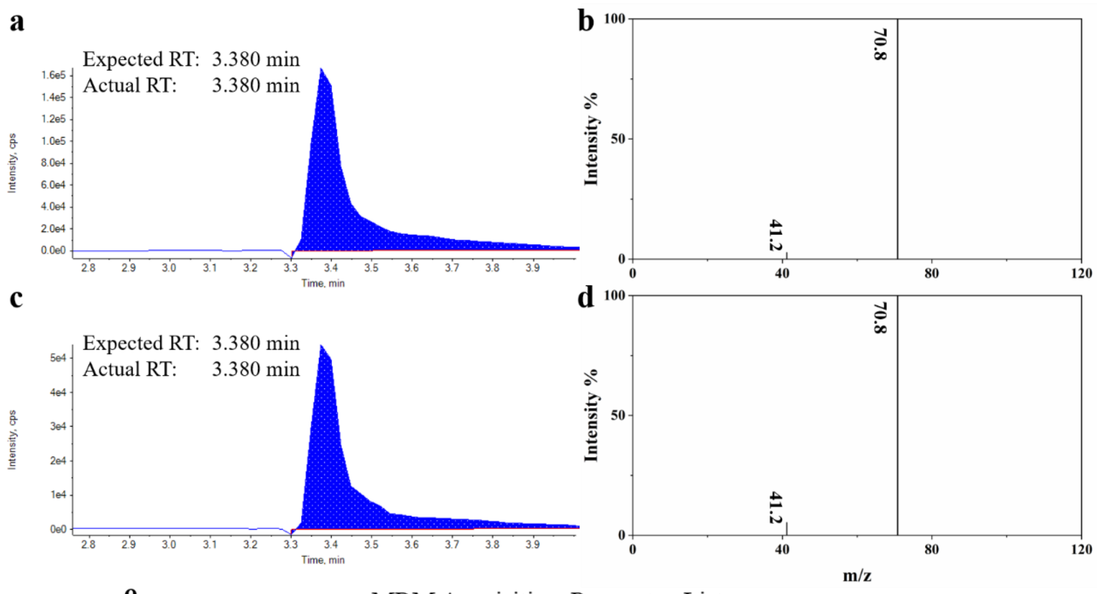

e

MRM Acquisition Parameter List

Analytes	Q1 Mass（Da）	Q3 Mass（Da）	DP(V)	CE(V)	CXP(V)
5-Hydroxymethyluracil	140.9	70.8	-100	-21	-5
	140.9	41.2	-100	-25	-7

Supplementary Figure 18. LC MS/MS of 5-hydroxymethyluracil. a) retention time in standard sample; b) MS/MS date in standard sample; c) retention time in serum sample; d) MS/MS data in serum sample; e) MRM acquisition parameter list.

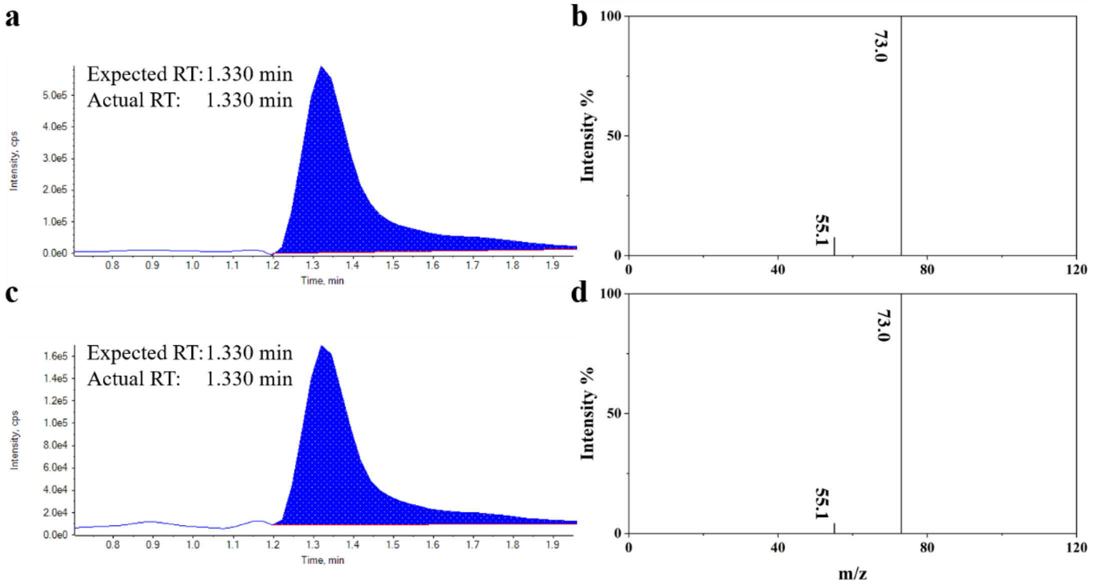

e

MRM Acquisition Parameter List

Analytes	Q1 Mass (Da)	Q3 Mass (Da)	DP(V)	CE(V)	CXP(V)
Methylmalonic acid	116.8	73.0	-60	-12	-4
	116.8	55.1	-60	-33	-7

Supplementary Figure 19. LC MS/MS of methylmalonic acid. a) retention time in standard sample; b) MS/MS date in standard sample; c) retention time in serum sample; d) MS/MS data in serum sample; e) MRM acquisition parameter list.

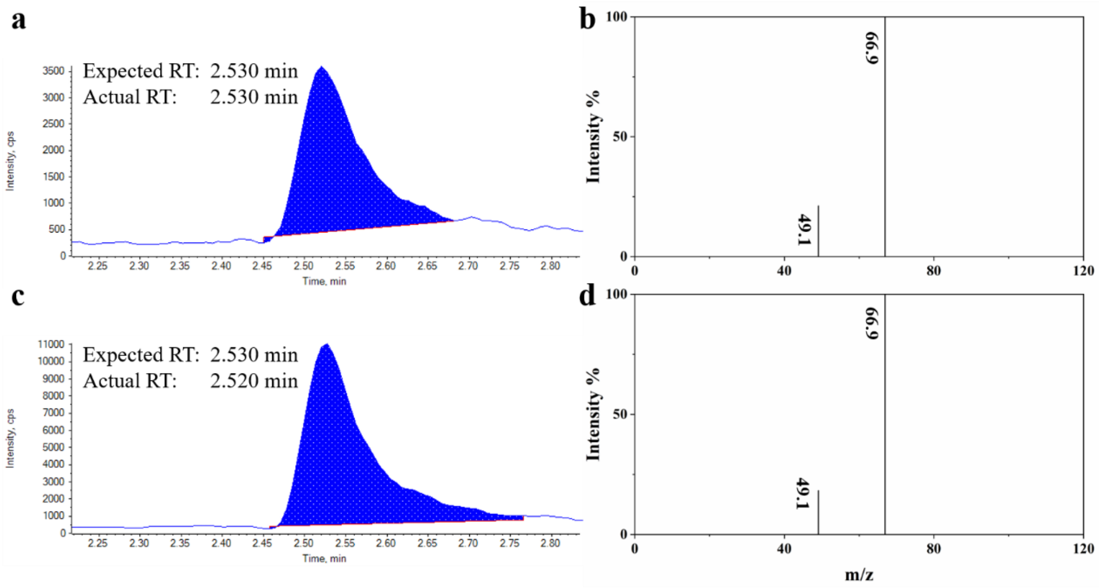

e

MRM Acquisition Parameter List

Analytes	Q1 Mass (Da)	Q3 Mass (Da)	DP(V)	CE(V)	CXP(V)
2-Furoic acid	111.0	66.9	-50	-15	-13
	111.0	49.1	-50	-36	-13

Supplementary Figure 20. LC MS/MS of 2-furoic acid. a) retention time in standard sample; b) MS/MS date in standard sample; c) retention time in serum sample; d) MS/MS data in serum sample; e) MRM acquisition parameter list.

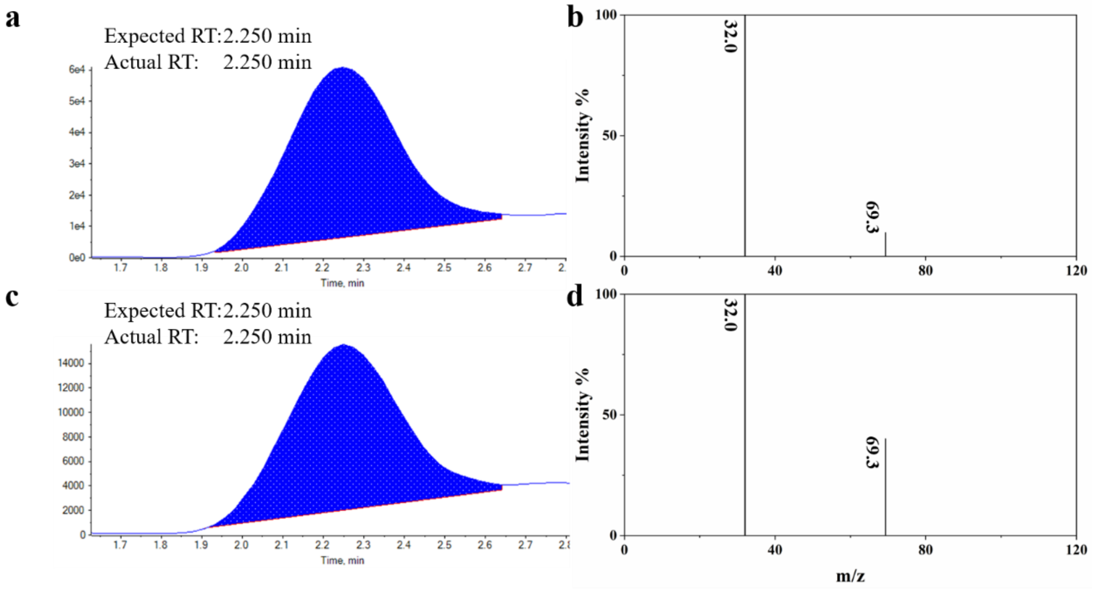

e

MRM Acquisition Parameter List

Analytes	Q1 Mass (Da)	Q3 Mass (Da)	DP(V)	CE(V)	CXP(V)
2-Oxovaleric acid	115.1	32.0	-50	-11	-3
	115.1	69.3	-50	-10	-4

Supplementary Figure 21. LC MS/MS of 2-oxovaleric acid. a) retention time in standard sample; b) MS/MS date in standard sample; c) retention time in serum sample; d) MS/MS data in serum sample; e) MRM acquisition parameter list.

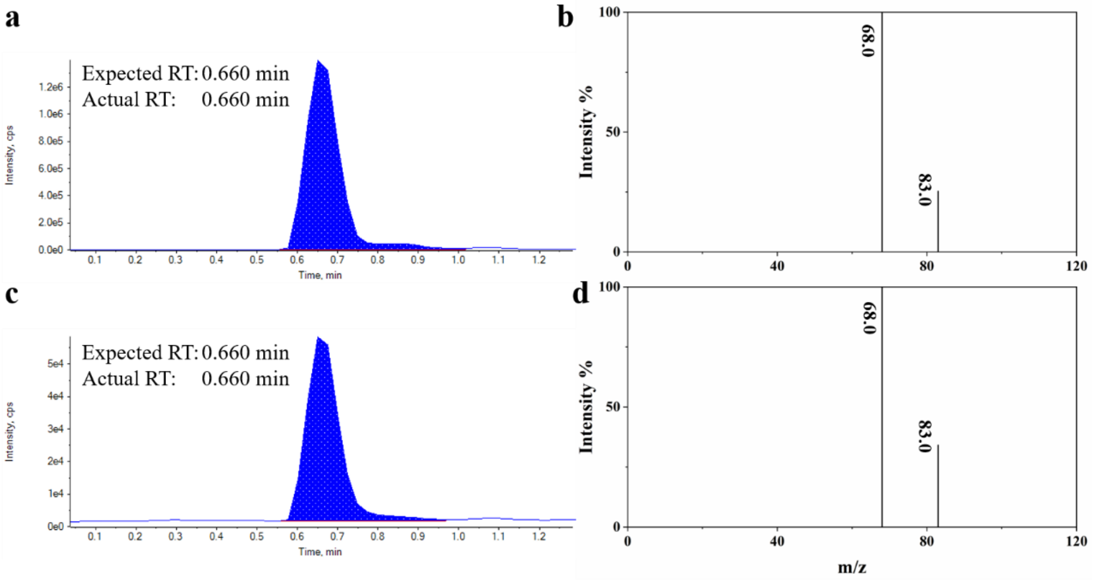

e MRM Acquisition Parameter List

Analytes	Q1 Mass（Da）	Q3 Mass（Da）	DP(V)	CE(V)	CXP(V)
Histamine	112.0	68.0	55	29	8
	112.0	83.0	57	20	5

Supplementary Figure 22. LC MS/MS of histamine. a) retention time in standard sample; b) MS/MS date in standard sample; c) retention time in serum sample; d) MS/MS data in serum sample; e) MRM acquisition parameter list.

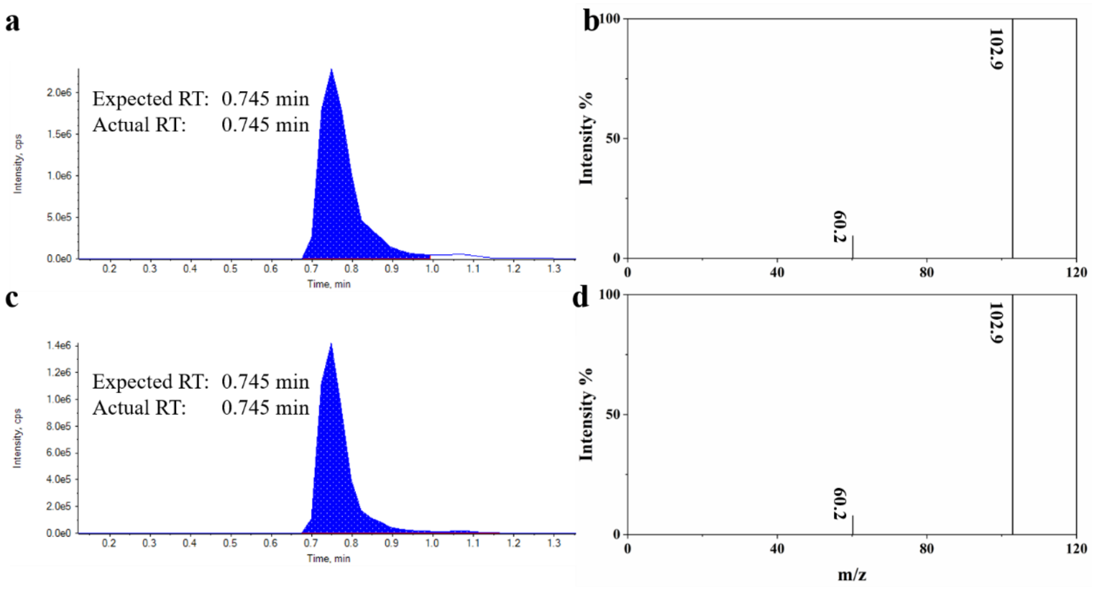

e

MRM Acquisition Parameter List

Analytes	Q1 Mass (Da)	Q3 Mass (Da)	DP(V)	CE(V)	CXP(V)
L-carnitine	161.6	102.9	80	22	6
	161.6	60.2	130	47	11

Supplementary Figure 23. LC MS/MS of L-carnitine. a) retention time in standard sample; b) MS/MS date in standard sample; c) retention time in serum sample; d) MS/MS data in serum sample; e) MRM acquisition parameter list.

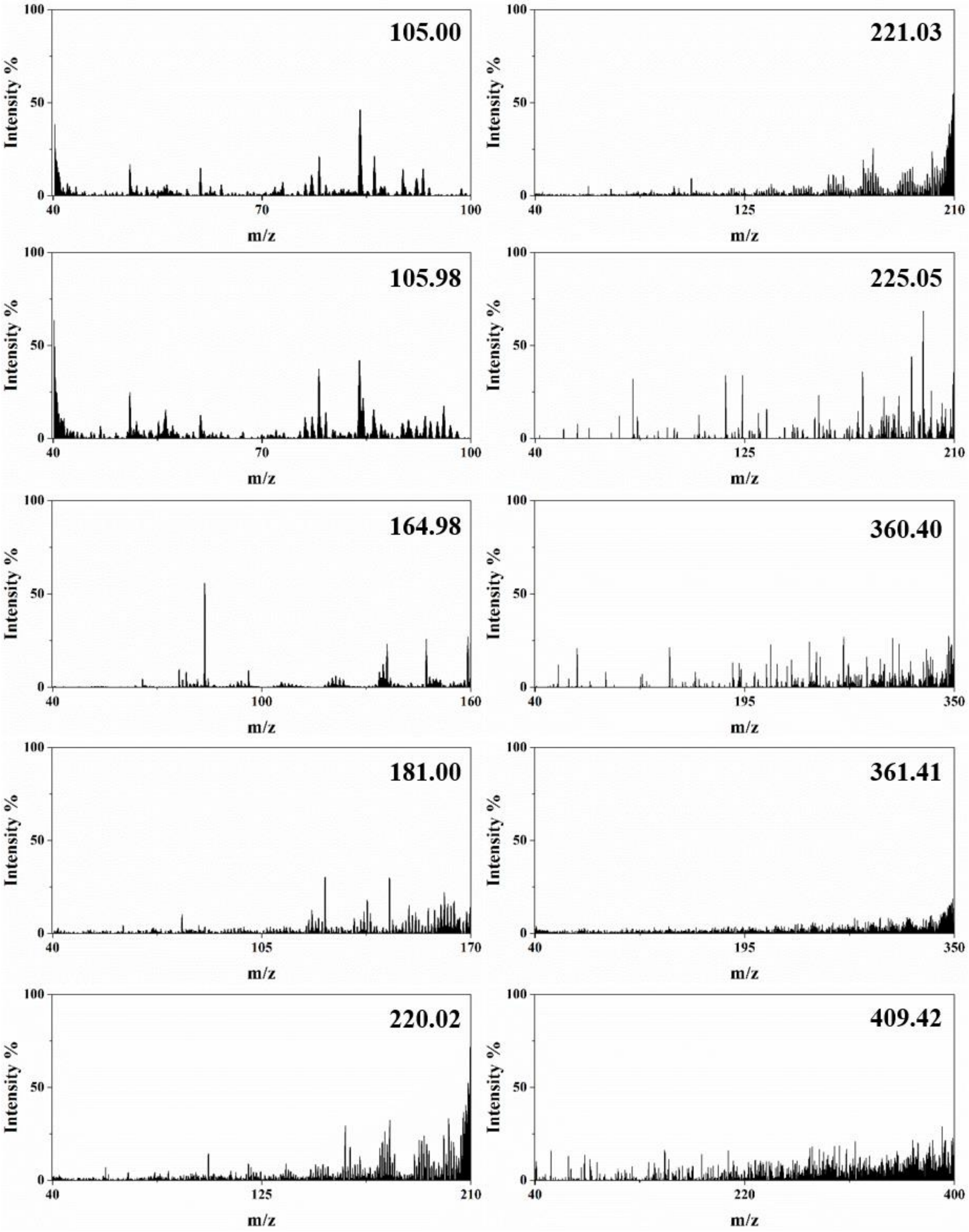

Supplementary Figure 24. MS/MS data of top 10 m/z of HCC in serum sample mixed with GNS.

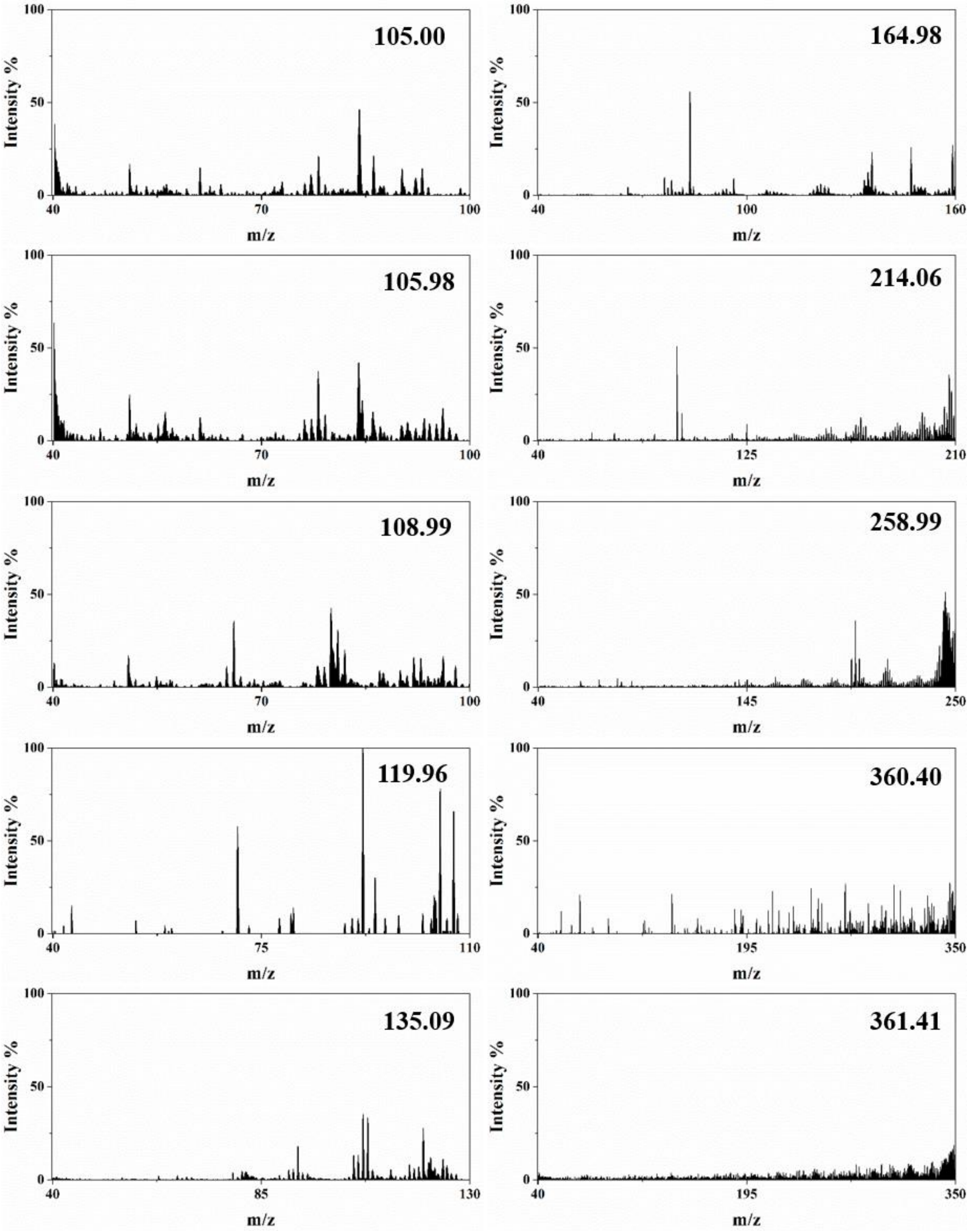

Supplementary Figure 25. MS/MS data of top 10 m/z of NSCLC in serum sample mixed with GNS.

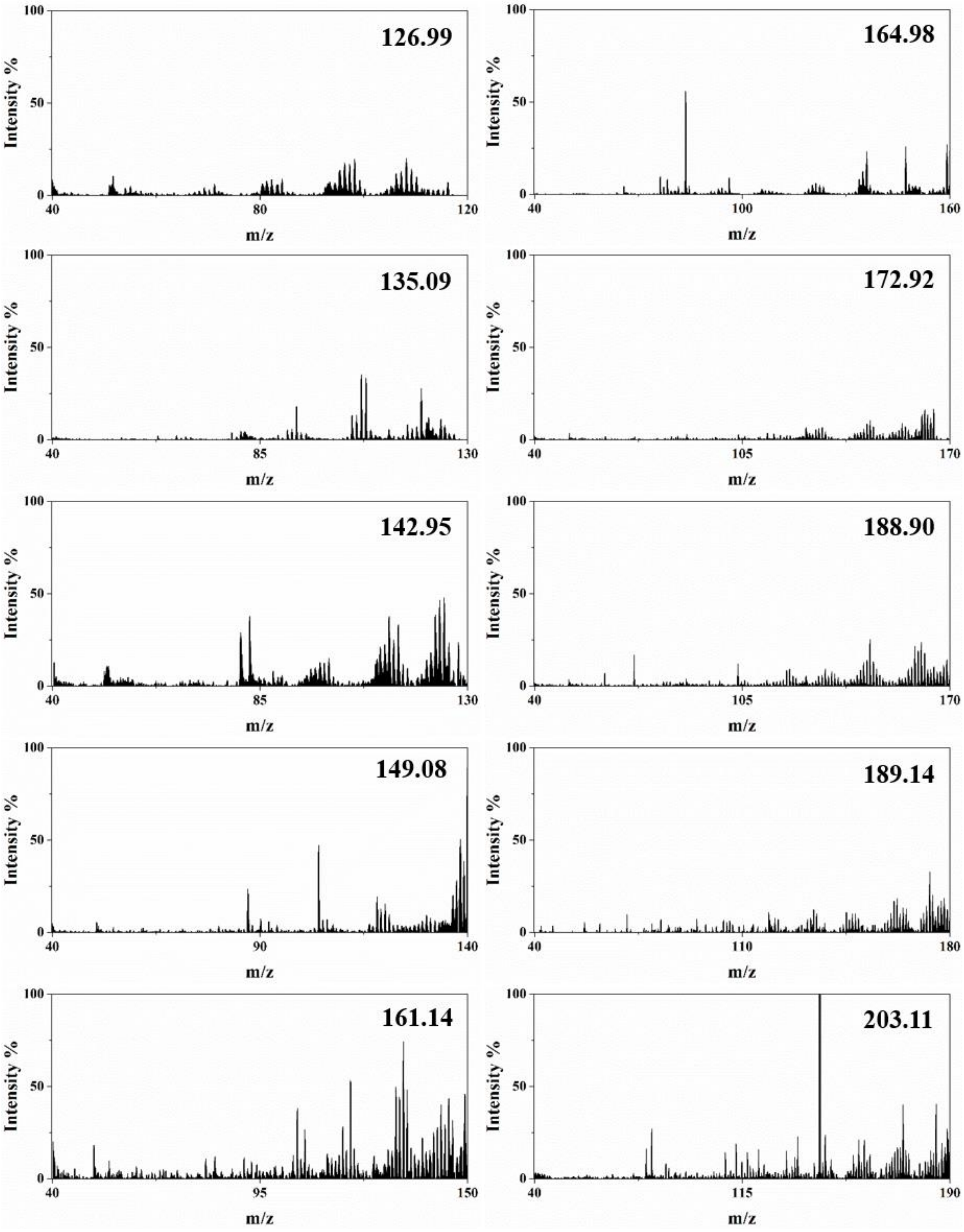

Supplementary Figure 26. MS/MS data of top 10 m/z of PAAD in serum sample mixed with GNS.

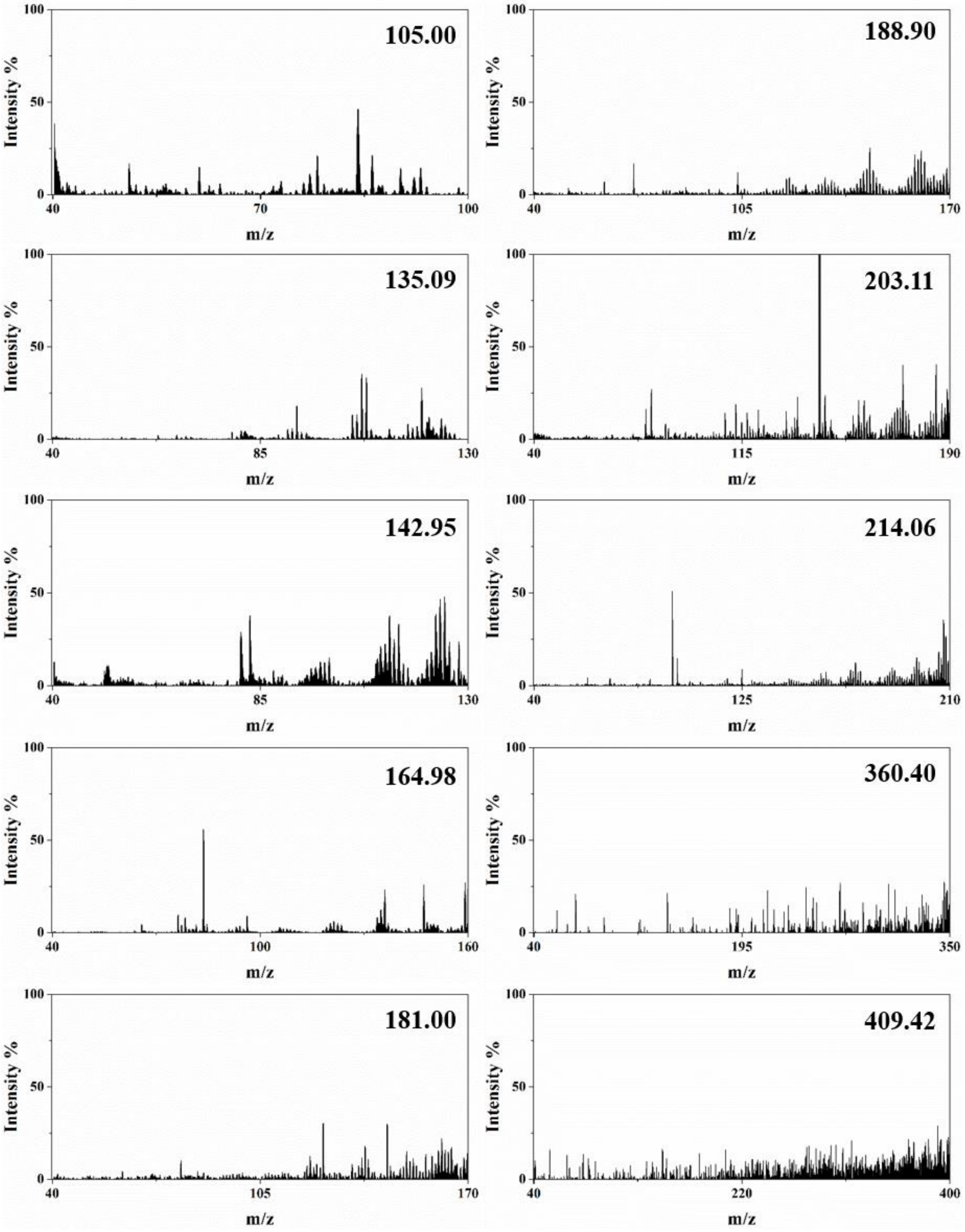

Supplementary Figure 27. MS/MS data of top 10 m/z of CRC in serum sample mixed with GNS.

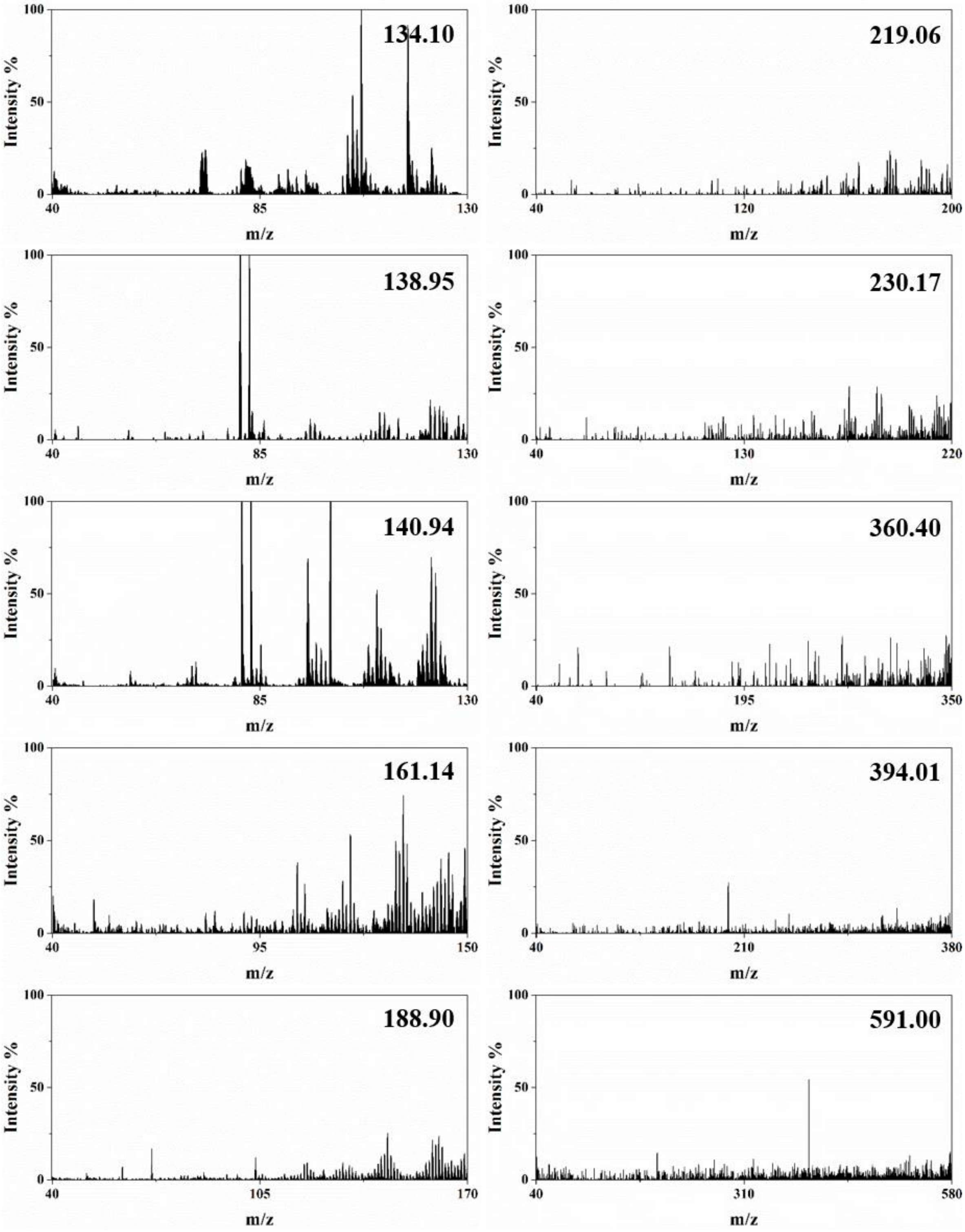

Supplementary Figure 28. MS/MS data of top 10 m/z of GC in serum sample mixed with GNS.

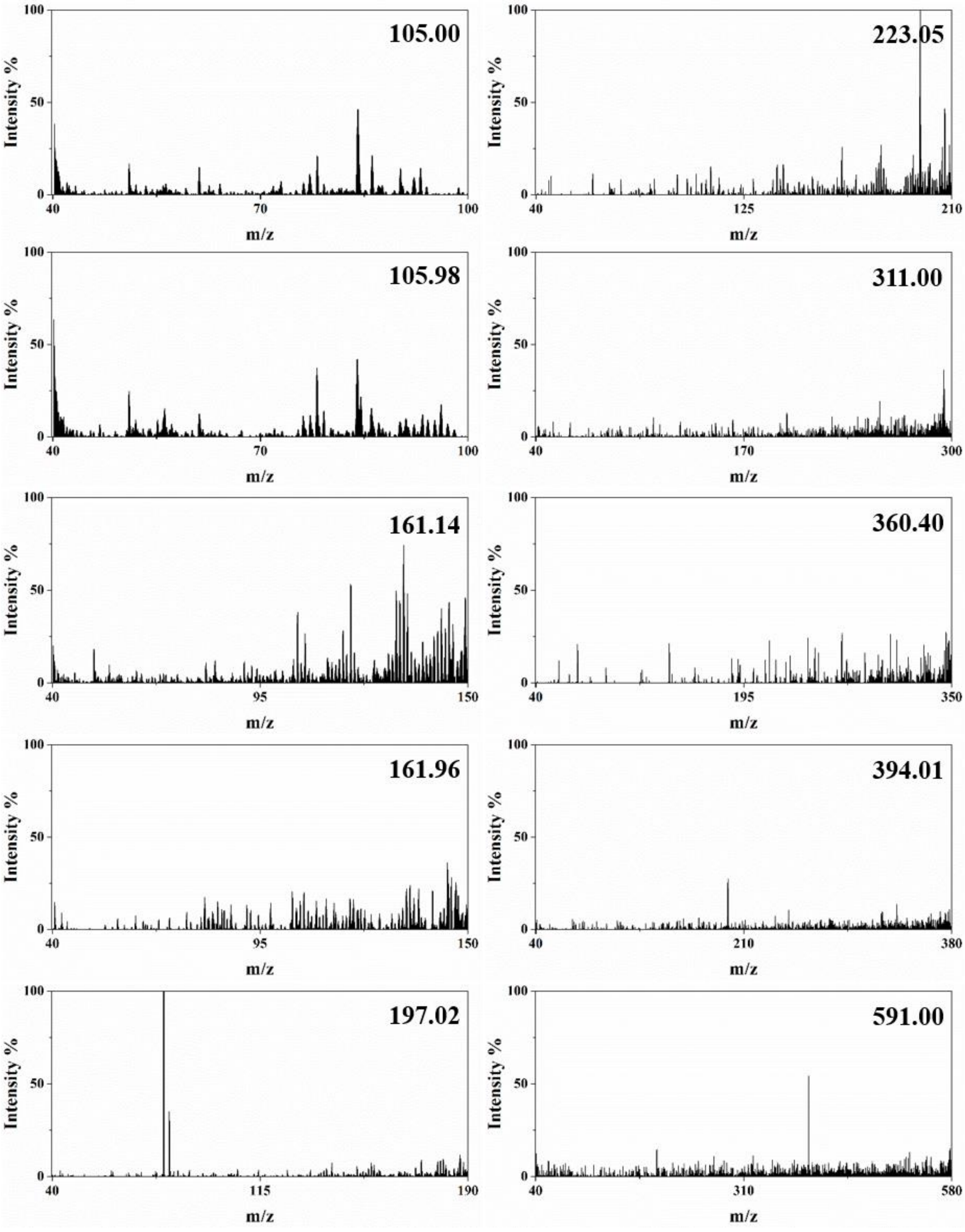

Supplementary Figure 29. MS/MS data of top 10 m/z of PTC in serum sample mixed with GNS.

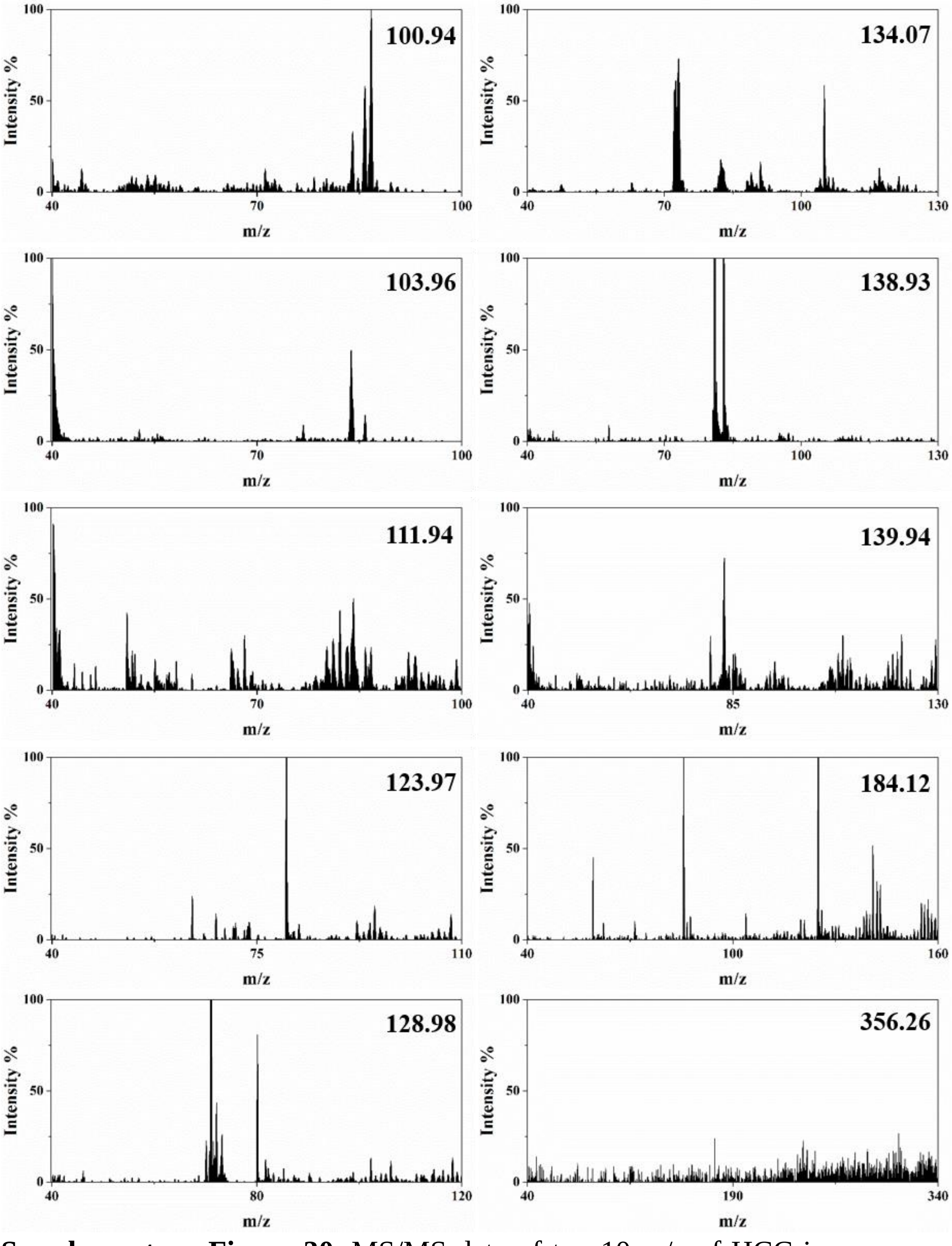

Supplementary Figure 30. MS/MS data of top 10 m/z of HCC in serum sample mixed with SiNW.

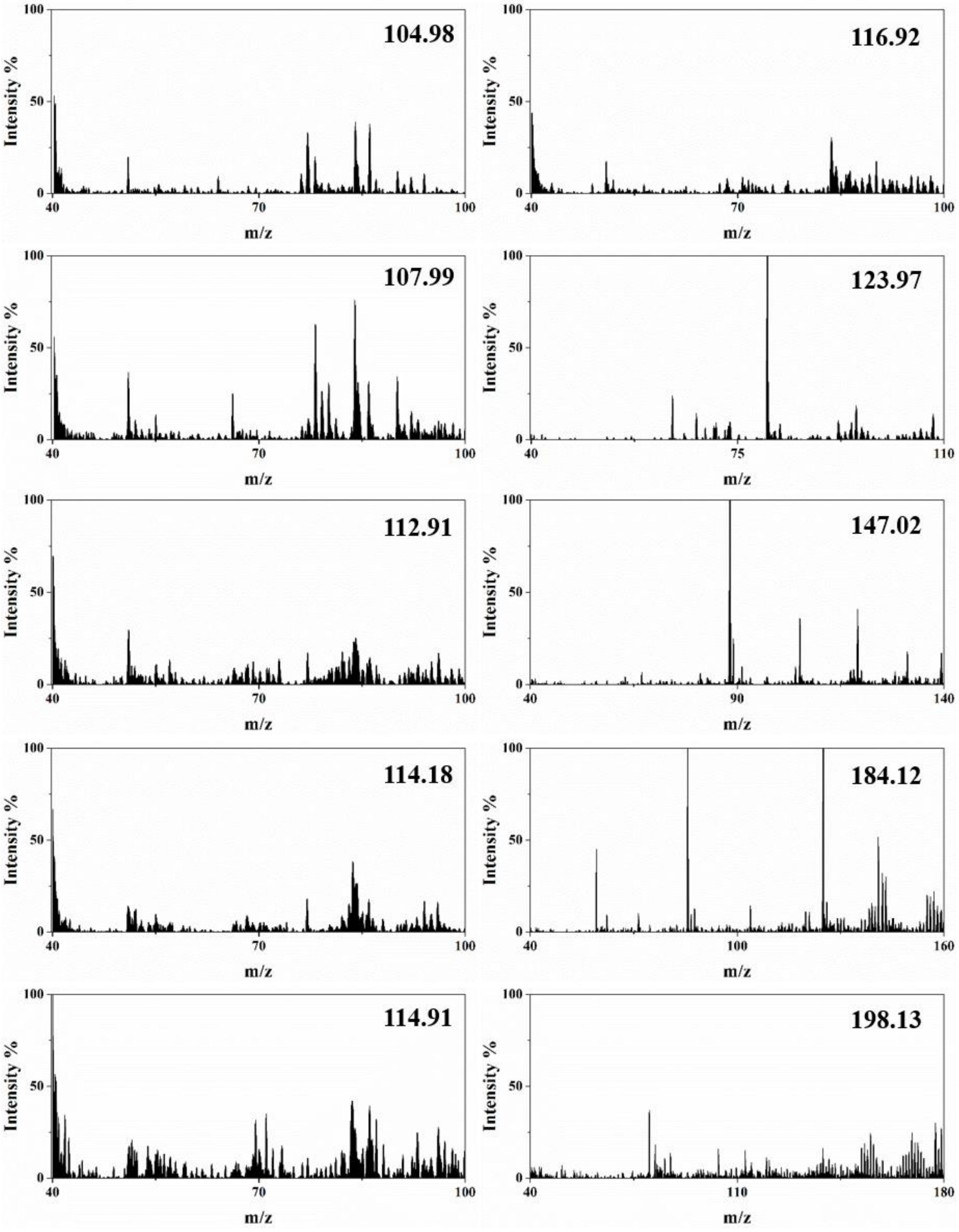

Supplementary Figure 31. MS/MS data of top 10 m/z of NSCLC in serum sample mixed with SiNW.

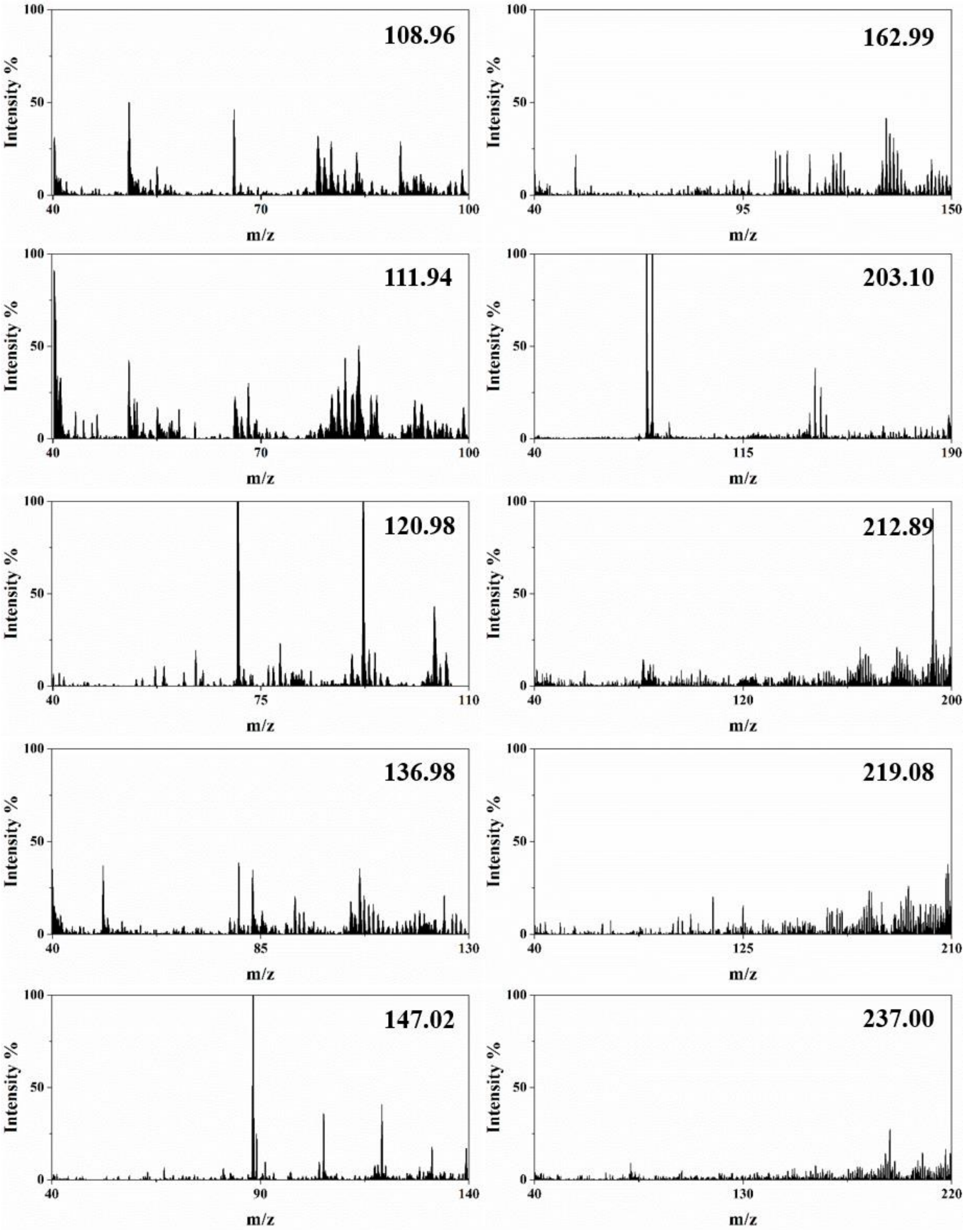

Supplementary Figure 32. MS/MS data of top 10 m/z of PAAD in serum sample mixed with SiNW.

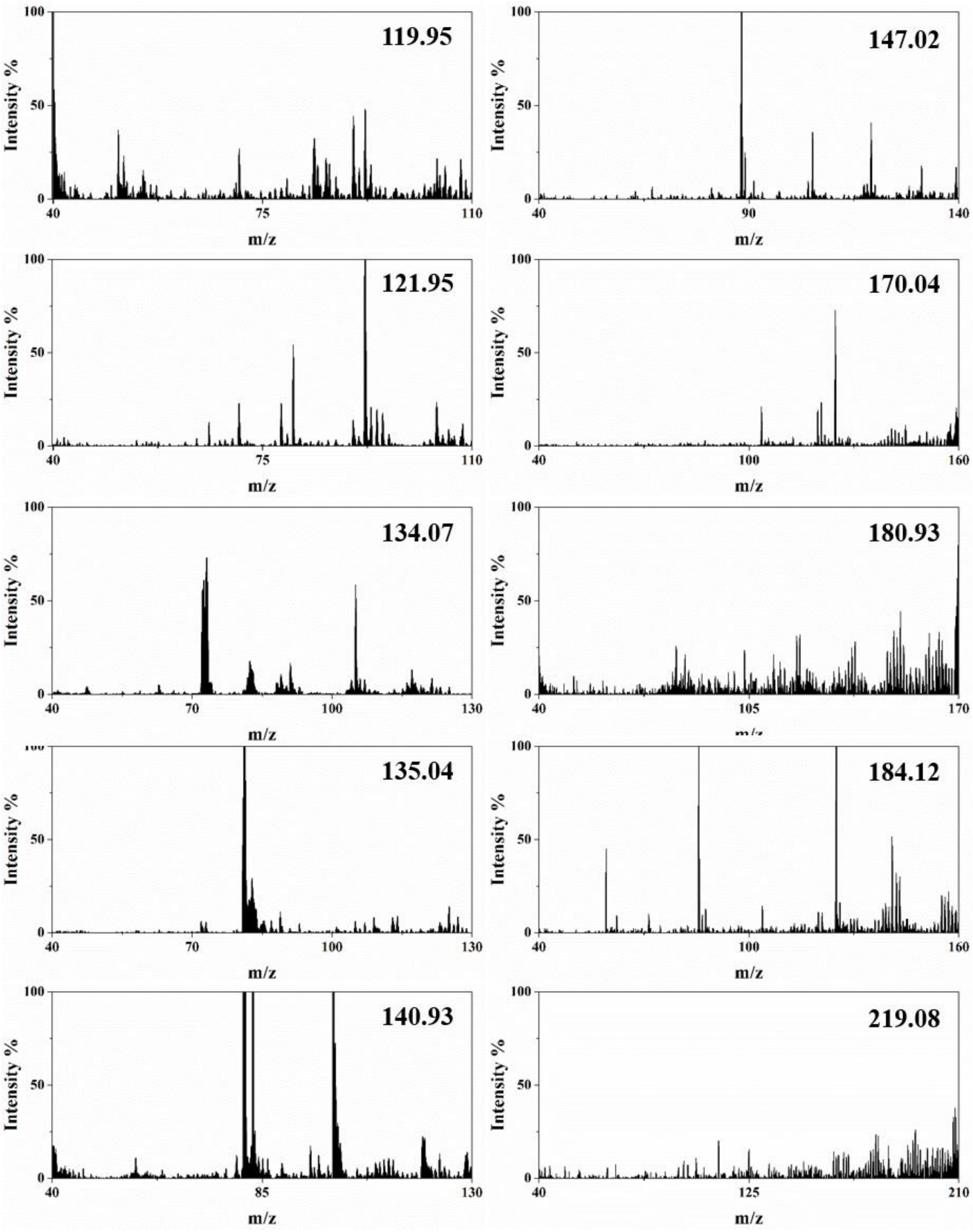

Supplementary Figure 33. MS/MS data of top 10 m/z of CRC in serum sample mixed with SiNW.

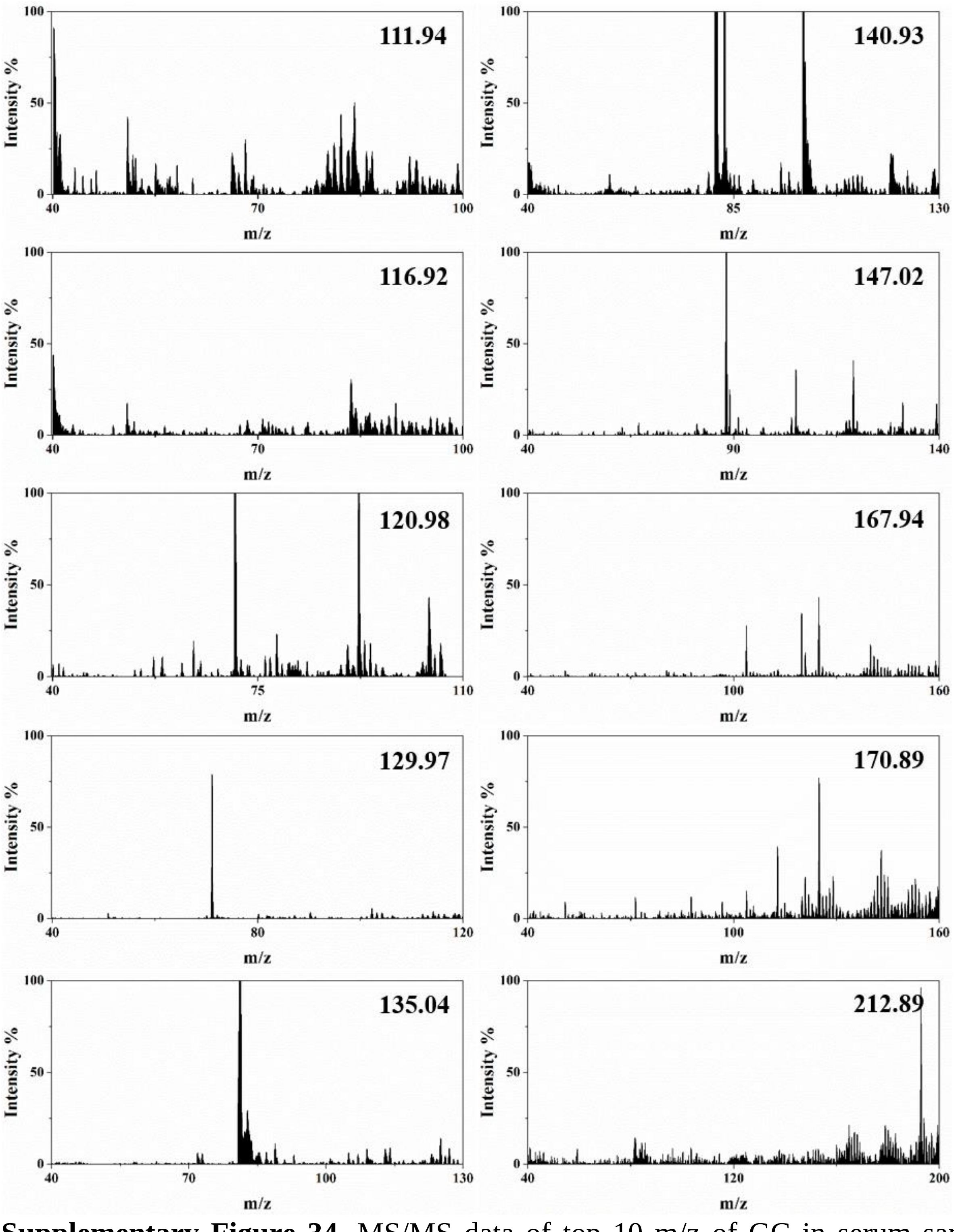

Supplementary Figure 34. MS/MS data of top 10 m/z of GC in serum sample mixed with SiNW.

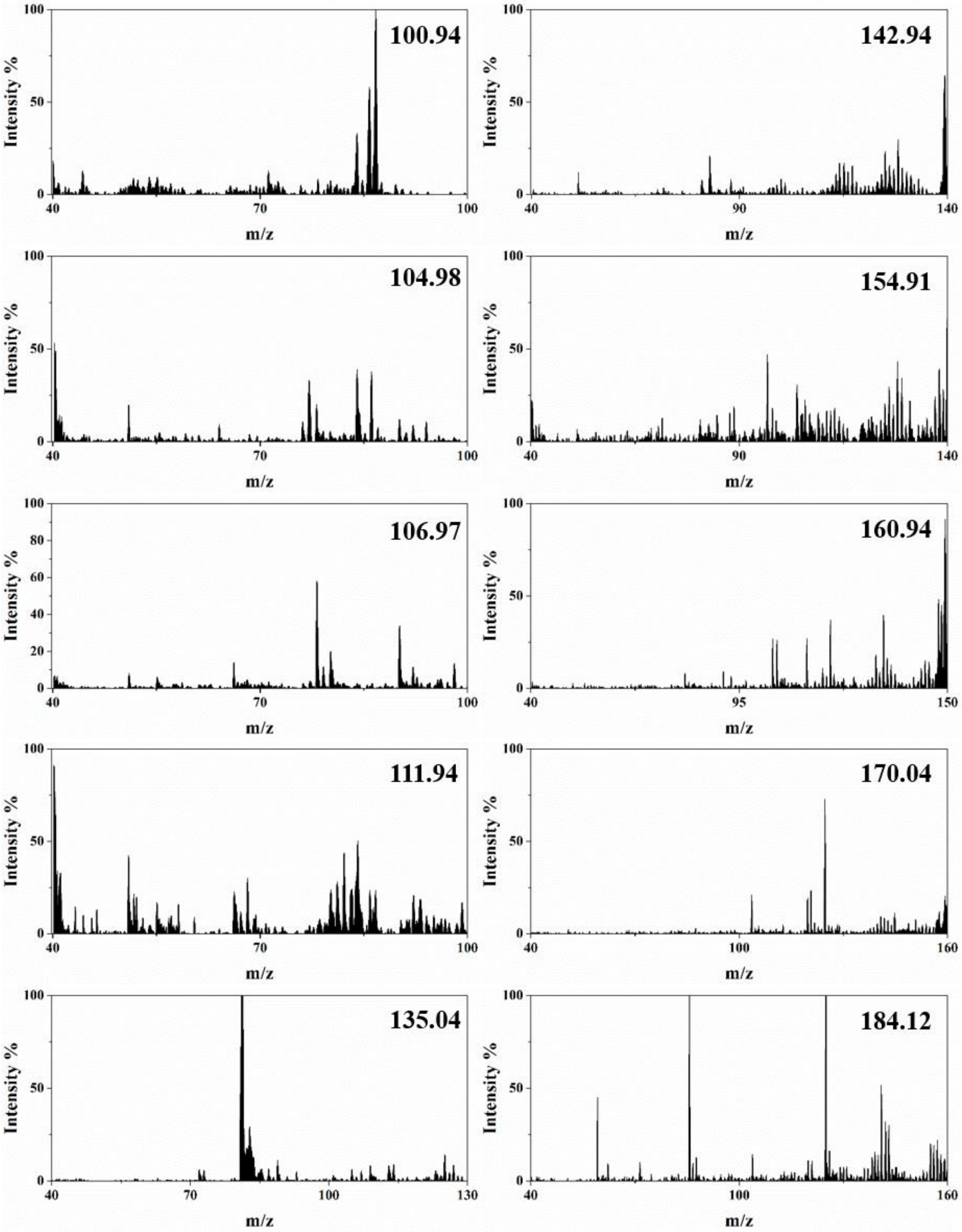

Supplementary Figure 35. MS/MS data of top 10 m/z of PTC in serum sample mixed with SiNW.

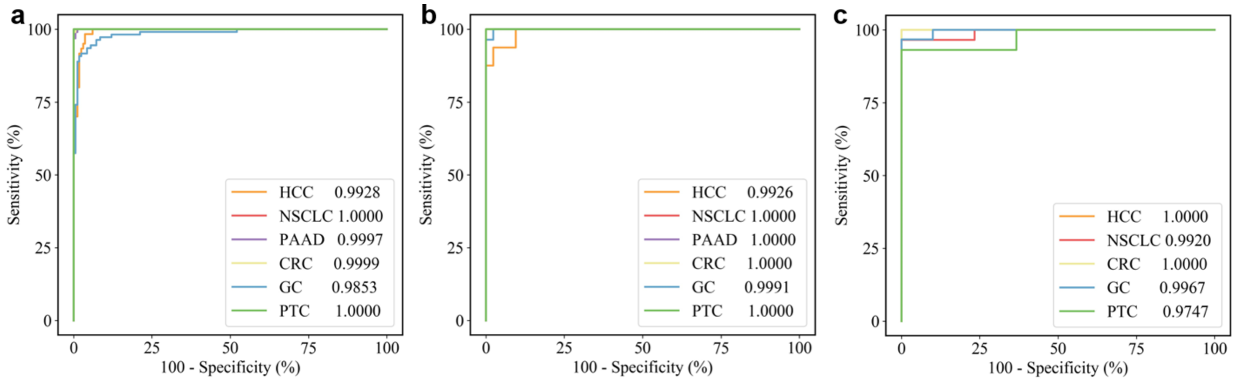

Supplementary Figure 36. ROC curves of each cancer type versus healthy controls in the training cohort (a), the internal (b) and the external (c) validation cohort.

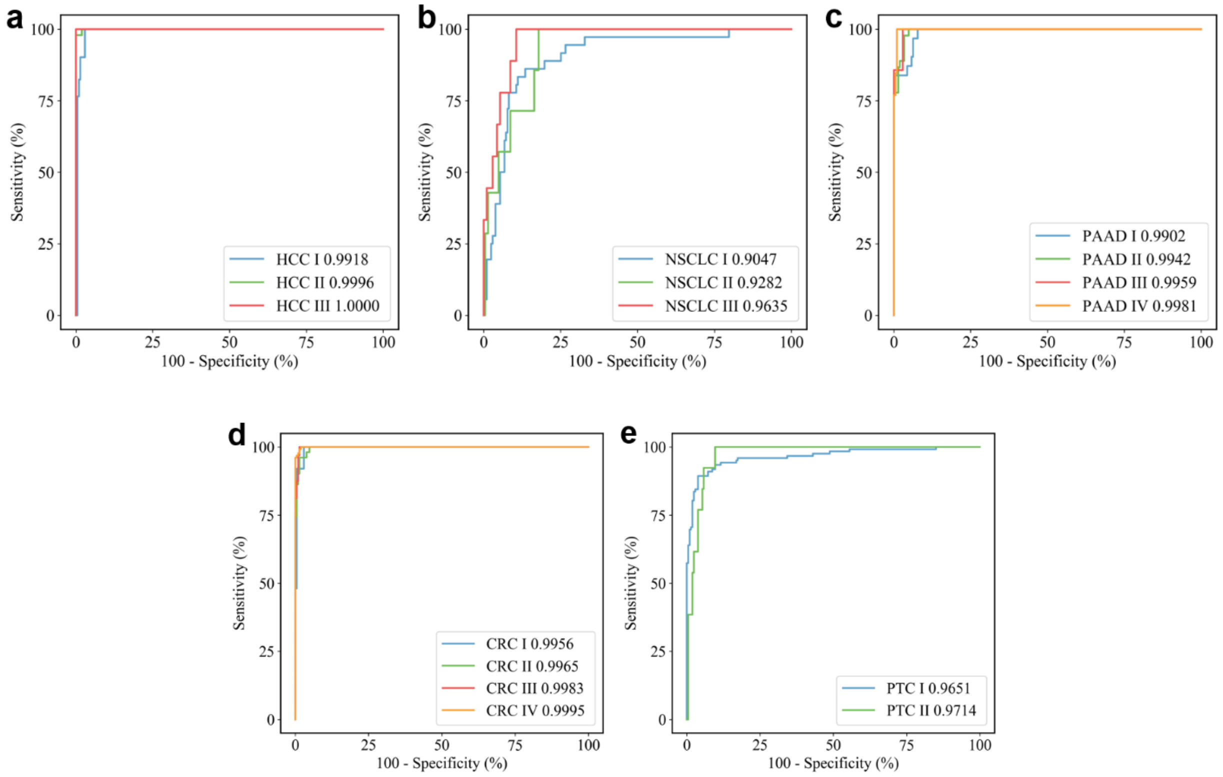

Supplementary Figure 37. ROC curves of different stages of HCC (a), NSCLC (b), PAAD (c), CRC (d) and PTC (e).

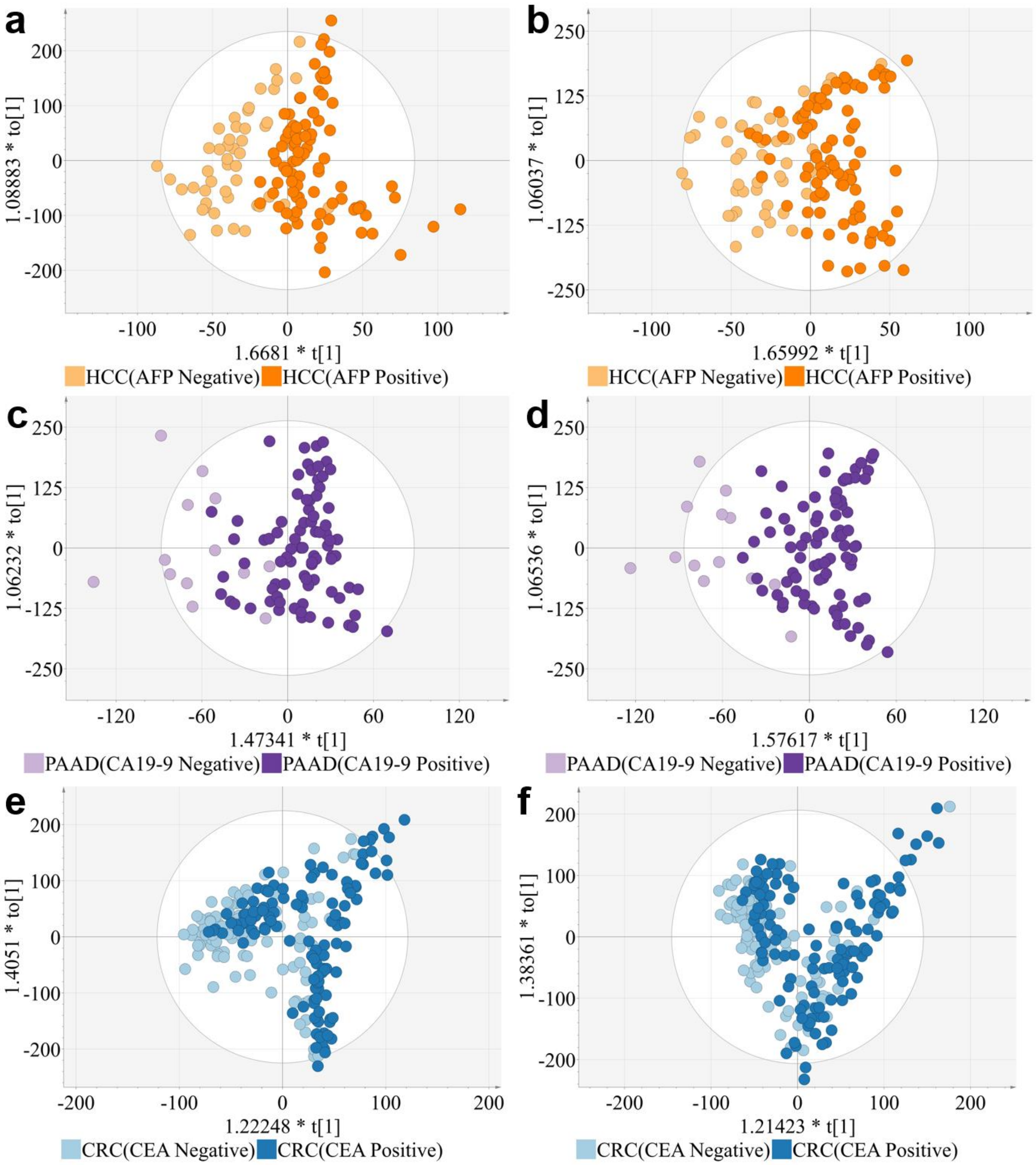

Supplementary Figure 38. OPLS-DA plots using SIMCA-P13. Input positive data and negative data with the label. OPLS-DA plots between AFP positive HCCs and AFP negative HCCs of GNS (a) and SiNW (b) assisted LDI. OPLS-DA plots between CA19-9 positive PAADs and CA19-9 negative PAADs of GNS (c) and SiNW (d) assisted LDI. OPLS-DA plots between CEA positive CRCs and CEA negative CRCs of GNS (e) and SiNW (f) assisted LDI.

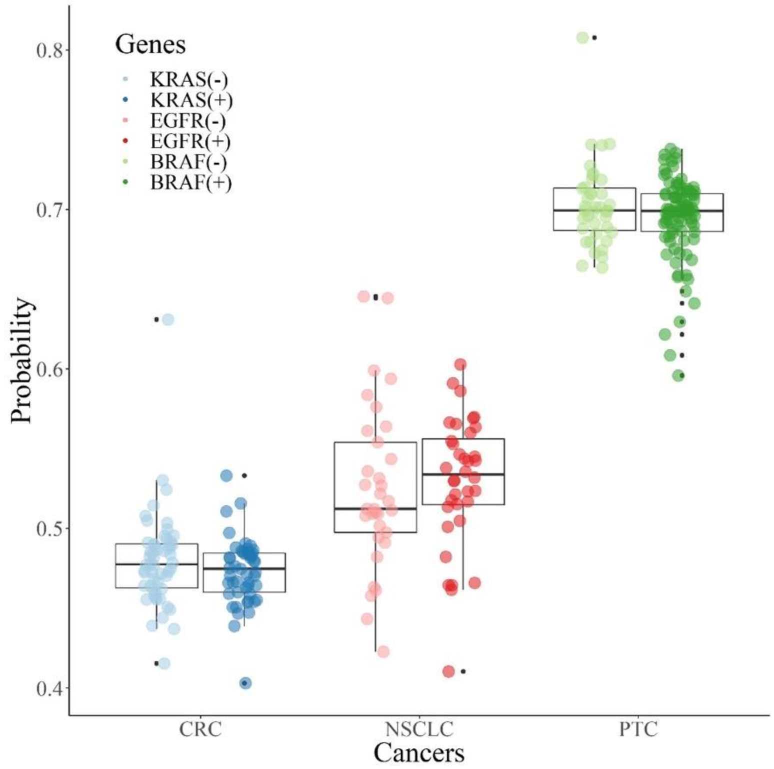

Supplementary Figure 39. X axis shows 49 of 103 CRC had KRAS(+), 36 of 69 NSCLC tumors had EGFR(+), and 91 of 130 PTC had BRAF(+), respectively while Y axis represents MNALCI output, which indicates that MNALCI was unable to discriminate common pathogenic mutations in CRC, NSCLC and PTC. The p-values by F-test were 0.275 for KRAS, 0.263 for EGFR, and 0.992 for BRAF, respectively. The probability shows no significant difference between mutation positive and negative tumors. In boxplots, center line, box edges and whiskers indicate the median, upper and lower quartiles (the 75th and 25th percentiles) and 1.5 × interquartile range, respectively. The different colors (blue, red and green) represented the three cancers CRC, NSCLC and PTC, respectively.

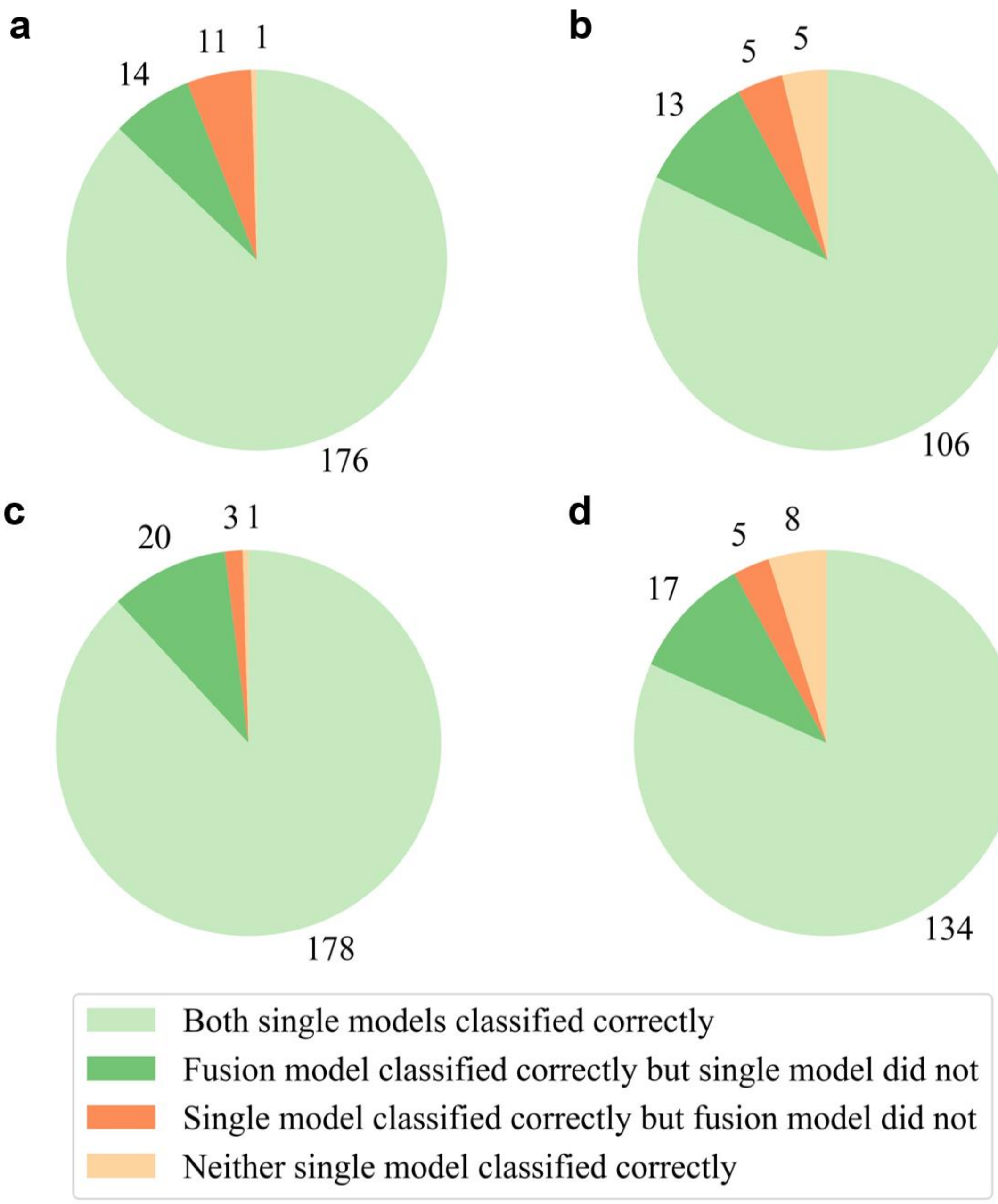

Supplementary Figure 40. Internal validation test using a cohort including 162 patients and 40 healthy controls, showing the results of the binary classification (a and c) and the multi-cancer classification (b and d). The thresholds were set at 1.5 (a and b) and 1.0 (c and d), respectively.
