# Supplementary material for: Multiplexed nanomaterial-assisted laser desorption/ionization for pan-cancer diagnosis and classification
Source: Nat Commun. 2022 Feb 1;13:617. doi: 10.1038/s41467-021-26642-9 (PMC8807648; doi:10.1038/s41467-021-26642-9)
Supplement: Supplementary file 2 — Reporting Summary [file 41467_2021_26642_MOESM2_ESM.pdf]

nature research

Corresponding author(s): Hua Wang, Yunbing Wang, Jingwei Bai, Xiaoying Li, Xiangfeng Duan

Last updated by author(s): Aug 1, 2021

Reporting Summary

Nature Research wishes to improve the reproducibility of the work that we publish. This form provides structure for consistency and transparency in reporting. For further information on Nature Research policies, see Authors & Referees and the Editorial Policy Checklist .

Statistics

For all statistical analyses, confirm that the following items are present in the figure legend, table legend, main text, or Methods section.

n/a	Confirmed	
☐	☒	The exact sample size (*n*) for each experimental group/condition, given as a discrete number and unit of measurement
☐	☒	A statement on whether measurements were taken from distinct samples or whether the same sample was measured repeatedly
☐	☒	The statistical test(s) used AND whether they are one- or two-sided<br>*Only common tests should be described solely by name; describe more complex techniques in the Methods section.*
☒	☐	A description of all covariates tested
☒	☐	A description of any assumptions or corrections, such as tests of normality and adjustment for multiple comparisons
☐	☒	A full description of the statistical parameters including central tendency (e.g. means) or other basic estimates (e.g. regression coefficient) AND variation (e.g. standard deviation) or associated estimates of uncertainty (e.g. confidence intervals)
☐	☒	For null hypothesis testing, the test statistic (e.g. *F*, *t*, *r*) with confidence intervals, effect sizes, degrees of freedom and *P* value noted<br>*Give P values as exact values whenever suitable.*
☒	☐	For Bayesian analysis, information on the choice of priors and Markov chain Monte Carlo settings
☒	☐	For hierarchical and complex designs, identification of the appropriate level for tests and full reporting of outcomes
☒	☐	Estimates of effect sizes (e.g. Cohen's *d*, Pearson's *r*), indicating how they were calculated

Our web collection on statistics for biologists contains articles on many of the points above.

Software and code

Policy information about availability of computer code

Data collection	All MS measurements were performed on an Autoflex Max mass spectrometer (Bruker Daltonics, Bremen, Germany), within a mass range of 100 to 1,000 Da, while the spectra were collected by FlexAnalysis 3.4 software (Bruker Daltonics, Bremen, Germany).
Data analysis	The authors used MALDIquantForeign(v0.12) package in R(v3.4.4) to convert the original data from mzml format to csv format. The main program is written in Python 3.7.3. Numpy(v1.17.2) and Pandas(v0.25.1) were used to process the data. The SVM model and related data processing functions were solved by the scikit-learn(v0.21.3) and scipy(v1.3.1). The confusion matrix, scatter diagram and ROC diagram are drawn by matplotlib (v3.1.1). The violin diagram is drawn by seaborn(v0.9.0).

For manuscripts utilizing custom algorithms or software that are central to the research but not yet described in published literature, software must be made available to editors/reviewers. We strongly encourage code deposition in a community repository (e.g. GitHub). See the Nature Research guidelines for submitting code & software for further information.

Data

Policy information about availability of data

All manuscripts must include a data availability statement. This statement should provide the following information, where applicable:

- Accession codes, unique identifiers, or web links for publicly available datasets
- A list of figures that have associated raw data
- A description of any restrictions on data availability

Source data are provided with this paper. The complete data that support the findings of this study are available from the corresponding author for research purpose only and the request will generally be answered to within 2 weeks. The python source code in this study can be found at https://github.com/zhengjiewhu/MNALCI.

Field-specific reporting

Please select the one below that is the best fit for your research. If you are not sure, read the appropriate sections before making your selection.

- [x] Life sciences
- [ ] Behavioural & social sciences
- [ ] Ecological, evolutionary & environmental sciences

For a reference copy of the document with all sections, see nature.com/documents/nr-reporting-summary-flat.pdf

Life sciences study design

All studies must disclose on these points even when the disclosure is negative.

Sample size	We did not predetermine the sample size but used group sizes typically for this type of work on basis of previous experiments using similar methodologies.<br>Ref:1. Cohen et al., Science 359, 926–930 (2018)<br>2.Cancer Cell. 2015 Nov 9;28(5):666-676.
Data exclusions	No data were excluded from analyses.
Replication	Experimental findings were independently reproduced by individual experiments and personnel.
Randomization	Pathological matched samples were assigned to experiment groups.
Blinding	No blinding was done in training cohort, but to evaluate the accuracy of MNALCI, we first tested with the single-blinded internal validation cohort(Shanghai cohort),then we tested with another single-blinded external validation cohort (Hefei cohort).

Reporting for specific materials, systems and methods

We require information from authors about some types of materials, experimental systems and methods used in many studies. Here, indicate whether each material, system or method listed is relevant to your study. If you are not sure if a list item applies to your research, read the appropriate section before selecting a response.

Materials & experimental systems

n/a	Involved in the study	
x		Antibodies
x		Eukaryotic cell lines
x		Palaeontology
x		Animals and other organisms
	x	Human research participants
x		Clinical data

Methods

n/a	Involved in the study	
x		ChIP-seq
x		Flow cytometry
x		MRI-based neuroimaging

Human research participants

Policy information about studies involving human research participants

Population characteristics	The Shanghai cohort composed of 1,008 individuals that include 203 healthy controls(age:23-76; Female 86;Male 117) and 805 patients(age 21-84; Female 328;Male 477) diagnosed with stage I to IV cancers according to American Joint Commission on Cancer (AJCC):liver cancer (n=139), lung cancer (n=76), pancreatic cancer (n=97), colorectal cancer (n=238), stomach cancer (n=119) and thyroid cancer (n=136) from Zhongshan Hospital, Fudan University in Shanghai, China.<br>The Hefei cohort including 175 individuals that include 30 healthy controls (age: 31-52; Female 8,Male 22)and 145 patient(age: 24-84;Female 68,Male 77) with stage I to IV cancers: liver cancer (n=29), lung cancer (n=28), colorectal cancer (n=30), stomach cancer (n=30), thyroid cancer (n=28) from the First Affiliated Hospital of Anhui Medical University in Hefei, China was further investigated as an external validation cohort in the present study.
Recruitment	The participants were recruited according to medical history. All cancer diagnoses were based on pathology. The inclusion criteria were (1) age ≥18 years; (2) agreement to participate in the data collection and serum sample collection before cancer treatment;(3)have complete clinical records. All the serum samples were collected before treatment, including surgery, chemotherapy, radiotherapy, etc. All samples were anonymized, and only the gender, age and cancer-related lab results and pathological diagnosis were recorded.<br>The healthy control serum samples in the training and internal validation cohort were collected at the Medical Examination Center (MEC) of Zhongshan Hospital Fudan University and control samples in the external validation cohort were collected at the MEC of the First Affiliated Hospital of Anhui Medical University. All healthy controls had normal biochemical profiles (including serum tumor antigens), negative ultrasound/radiological findings and no previous history of any type of cancer.<br>Limitation: First, the sample size of the external validation cohort was small and without PAAD patients. Future studies should ideally include multi-center blinded cohorts with larger size and comparable composition of cancers for validation. Secondly, for each type of cancer, the total number of patients included was relatively small for machine learning. If more cancer types were included, the accuracy for classifying each individual cancer could be compromised. Nevertheless, the accuracy of MNALCI is expected to increase if more high-quality data are available for training.
Ethics oversight	The study was approved by the Ethics Committee of Zhongshan Hospital Fudan University and the First Affiliated Hospital of Anhui Medical University.

Note that full information on the approval of the study protocol must also be provided in the manuscript.
