# Supplementary material for: Multiplexed nanomaterial-assisted laser desorption/ionization for pan-cancer diagnosis and classification
Source: Nat Commun. 2022 Feb 1;13:617. doi: 10.1038/s41467-021-26642-9 (PMC8807648; doi:10.1038/s41467-021-26642-9)
Supplement: Supplementary file 4 — Description of Additional Supplementary Files [file 41467_2021_26642_MOESM4_ESM.docx]

Legends for Supplementary Data for

**Multiplexed Nanomaterial-Assisted Laser Desorption/Ionization for Pan-cancer Diagnosis and Classification**

Supplementary Data 1: Comparison of Threshold Selection Results Based on Training Cohort

Supplementary Data 2: P Value of Top 10 Discriminative m/z Features of Each Cancer Type vs Healthy Control (F-test was used to calculate the p-values)

Supplementary Data 3: Single Blind Internal Validation Test Using a Cohort including 161 Patients and 41 Healthy Controls (Threshold=1)

Supplementary Data 4: Single Blind External Validation Test Using a Cohort including 145 Patients and 30 Healthy Controls (Threshold=1)

Supplementary Data 5: Single Blind Internal Validation Test Using a Cohort including 161 Patients and 41 Healthy Controls (Threshold=1.5)

Supplementary Data 6: Single Blind External Validation Test Using a Cohort including 145 Patients and 30 Healthy Controls (Threshold=1.5)

Supplementary Data 7: Six Patient Samples Exemplifying Different Situations of Pan-cancer Diagnosis and Classification (Binary Classification and Multi-class Classification)
